# Local and global genetic diversity of protozoan parasites: Spatial distribution of *Cryptosporidium* and *Giardia* genotypes

**DOI:** 10.1371/journal.pntd.0005736

**Published:** 2017-07-13

**Authors:** Juan C. Garcia–R, Nigel French, Anthony Pita, Niluka Velathanthiri, Rima Shrestha, David Hayman

**Affiliations:** 1 Molecular Epidemiology and Public Health Laboratory, Hopkirk Research Institute, Massey University, Palmerston North, New Zealand; 2 Institute of Veterinary, Animal & Biomedical Sciences, Massey University, Palmerston North, New Zealand; Yale University, UNITED STATES

## Abstract

Cryptosporidiosis and giardiasis are recognized as significant enteric diseases due to their long-term health effects in humans and their economic impact in agriculture and medical care. Molecular analysis is essential to identify species and genotypes causing these infectious diseases and provides a potential tool for monitoring. This study uses information on species and genetic variants to gain insights into the geographical distribution and spatial patterns of *Cryptosporidium* and *Giardia* parasites. Here, we describe the population heterogeneity of genotypic groups within *Cryptosporidium* and *Giardia* present in New Zealand using gp60 and *gdh* markers to compare the observed variation with other countries around the globe. Four species of *Cryptosporidium* (*C*. *hominis*, *C*. *parvum*, *C*. *cuniculus* and *C*. *erinacei*) and one species of *Giardia* (*G*. *intestinalis*) were identified. These species have been reported worldwide and there are not unique *Cryptosporidium* gp60 subtype families and *Giardia* *gdh* assemblages in New Zealand, most likely due to high gene flow of historical and current human activity (travel and trade) and persistence of large host population sizes. The global analysis revealed that genetic variants of these pathogens are widely distributed. However, genetic variation is underestimated by data biases (e.g. neglected submission of sequences to genetic databases) and low sampling. New genotypes are likely to be discovered as sampling efforts increase according to accumulation prediction analyses, especially for *C*. *parvum*. Our study highlights the need for greater sampling and archiving of genotypes globally to allow comparative analyses that help understand the population dynamics of these protozoan parasites. Overall our study represents a comprehensive overview for exploring local and global protozoan genotype diversity and advances our understanding of the importance for surveillance and potential risk associated with these infectious diseases.

## Author summary

Infectious diseases threaten the health and well-being of wildlife, livestock and human populations and contribute to significant economic impact in agriculture and medical care. *Cryptosporidium* and *Giardia* are enteric protozoan pathogens that cause diarrhea and nutritional disorders on a global level. Using molecular analysis and a review framework we showed that species and genetic variants within genera *Cryptosporidium* and *Giardia* (including two species recently infecting humans) found in an island system are not different from other parts of the world. This similarity is likely due to high gene flow of historical and current human activity (travel and trade) and persistence of large host population sizes, such as cattle and people. We also show that, although species and genotypes are widely distributed, new variants will arise when sampling effort increase and their dispersal will be facilitated by human activity. These findings suggest that geographical distribution of species and genotypes within *Cryptosporidium* and *Giardia* parasites may yield important clues for designing effective surveillance strategies and identification of factors driving within and cross species transmission.

## Introduction

Infectious diseases are the major leading causes of death and disability worldwide [1]. Classical public health and sanitation measures have long served to minimize dissemination and human exposure to many pathogens that are spread by routes such as contaminated water or via vectors [2] but the recent Ebola and Zika outbreaks have highlighted the need for a global framework to reduce the risk and mitigate health crises [3–5]. Determining genetic diversity and the distribution of microbes can guide more efficient interventions that decrease the incidence of infectious diseases. Understanding patterns of geographical distribution and genetic variation is essential to monitor pathogen evolution and adaptation, design effective surveillance strategies, identify factors driving within and cross species transmission, and ultimately, reduce the burden of disease [6–8].

Gastroenteritis is a well-known public health threat and a significant burden of disease that requires improved monitoring [2, 9]. A substantial number of gastroenteritis cases around the world are caused by the parasitic protozoans *Cryptosporidium* and *Giardia* [10–14]. These organisms are recognized as major causes of parasite-induced diarrhoea in humans and other animals that can be spread through various means, but transmitted mainly by interactions between humans and animals and by contaminated water or food [14]. Most human cryptosporidiosis cases worldwide are caused by *Cryptosporidium parvum* and *C*. *hominis* [15], although other species in domesticated and wild animals can infect humans and cause high morbidity and mortality in children, travellers and immunocompromised individuals [11, 16–21]. It has been suggested that *Cryptosporidium* can be found in 1% of stools of immunocompetent hosts from high-income countries and 5–10% of hosts from low-resource settings but recent molecular studies suggest an underestimation of the reported frequency of infection and the global burden of the disease [16, 22]. *Giardia* is a common enteric parasite that infects a wide range of mammals, including domestic animals (livestock, dogs and cats), birds and amphibians. In humans, *G*. *intestinalis* (synonyms *G*. *lamblia* and *G*. *duodenalis*) is the most common intestinal protozoan parasite, frequently reported in association with water- and food-borne outbreaks [15, 23, 24], affecting about 280 million people worldwide [25] with some 500,000 new symptomatic cases reported each year in developing countries only [26].

Species within each of these two protozoan genera are morphologically indistinguishable and their taxonomic and genotype differentiation is mainly based on molecular characterization [14, 27–29]. Multilocus sequence typing has been developed for identification and genotyping of *Cryptosporidium* and *Giardia* but the most commonly employed markers in epidemiological investigations and population studies are the cell surface glycoprotein (gp60) and glutamate dehydrogenase (*gdh*), respectively [30, 31]. The gp60 and *gdh* genes have been readily adopted as a key component for molecular epidemiological investigations because of their high reliability in the characterization of genotypes, standardization in genotype nomenclature and detection of variants within populations [15]. Although not all *Cryptosporidium* species can be detected with the gp60 typing tool [23, 32–34] a high variety of subtype families have been identified based on this gene, whilst several assemblages can be determined using *gdh* for *Giardia*.

**Fig 1:**
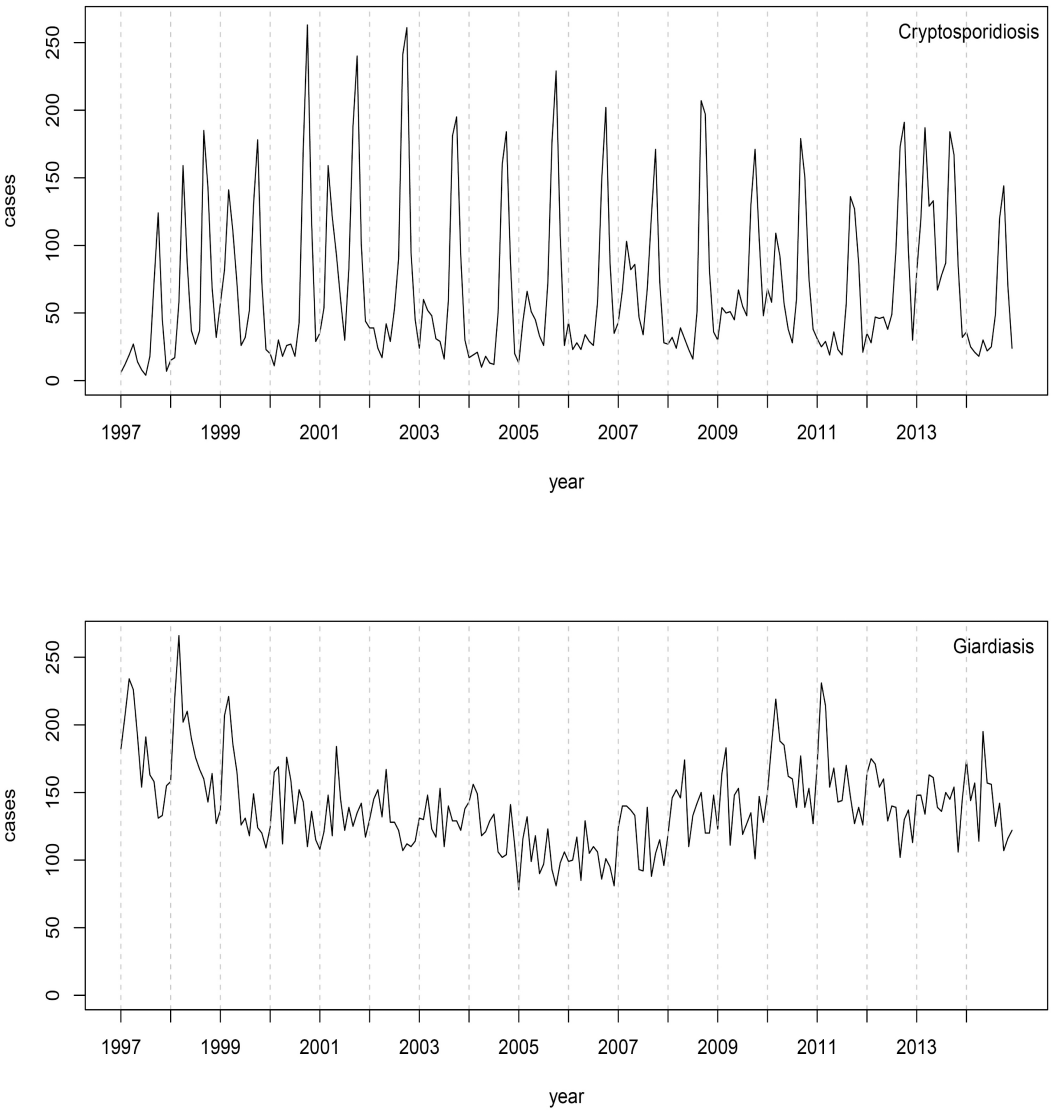
Trends in cryptosporidiosis and giardiasis notifications in New Zealand. Source: New Zealand Public Health Observatory (http://www.nzpho.org.nz/NotifiableDisease.aspx).

Recent studies have revealed that some genotypes are genetically diverse, host restricted and comprise a zoonotic or anthroponotic reservoir. For instance, *C*. *parvum* subtype families IIc and IIe are considered anthroponotic and IIa is predominant in humans and other animals worldwide [35] whilst *G*. *intestinalis* genotypes A and B are the only assemblages found in humans [23]. Additionally, the prevalence of some genotypes of *Cryptosporidium* and *Giardia* are unevenly distributed in different regions of the world [15, 36] with major occurrences of novel and rare variants in developing countries compared to developed countries [15, 30]. However, both diseases have been reported with a high incidence rate in New Zealand [15, 37]. The peak of cryptosporidiosis cases each year coincides with the cattle calving season [38, 39], while giardiasis incidence is more evenly distributed throughout the year (Fig 1). The annual number of notifications reported in New Zealand range between 500 and 700 and 1600 and 1800 for cryptosporidiosis and giardiasis, respectively [40]. Here, we reported the species and genotypes of *Cryptosporidium* and *Giardia* in New Zealand using gp60 and *gdh* markers during a long-term monitoring study (2009–2015). New Zealand has a recent and limited domestic animal movement [41] and it is an excellent island system example to compare genetic diversity against continental situations. Genotypes for species isolated from New Zealand were contrasted with those recovered from other countries to understand their spatial distribution and similarity. Specifically, we determined the genetic variants of gp60 subtype families in *Cryptosporidium* and *gdh* assemblages in *Giardia* to address the following questions: What are the subtype families of *Cryptosporidium* and assemblages of *Giardia* in New Zealand detected with gp60 and *gdh* genetic markers? How is the genetic variation of these parasites distributed in New Zealand compared to the rest of the world?

## Materials and methods

### Sampling

We performed an observational study of giardiasis and cryptosporidiosis in New Zealand. Both diseases are notifiable in New Zealand; therefore, suspected cases are confirmed through accredited laboratories. Fresh feacal samples were screened from symptomatic humans in New Zealand between 2009 and 2015 using either fluorescence microscopy or ELISA analysis in three national diagnostic laboratories, from both the North and South Islands of New Zealand. Positive stool samples were sent to the Hopkirk Research Institute, Massey University, New Zealand for further molecular typing analyses (see below). Human samples were submitted anonymously and no information about the patients was available. Our human sample covers 10% (range 4–19%) notified cryptosporidiosis and 13% (2–32%) notified giardiasis cases from 2009 to 2015 in New Zealand. A microscopy-based screen was employed for feacal samples of other sources that were obtained from farms, zoos, animal hospitals and urban wildlife, on an *ad hoc* basis, and as part of other studies [e.g., 42]. Stools were deposited in 2 ml screw cap tubes and stored at 4°C in the laboratory until DNA extraction.

### Molecular techniques

DNA extractions from stool samples were performed using the Isolate fecal DNA (Bioline) or QIAamp DNA Stool (Qiagen) kits following the manufacturer’s instructions and carried out at the Hopkirk Research Institute. DNA extraction required disruption of the oocyst/cysts using a beadbeater (Tissue Lyser II, Qiagen) at 30 Hz for 5 min. DNA quantification was measured using the Qubit fluorometer. A fragment of the gp60 and *gdh* genes were PCR amplified using a combination of external and internal primers (S1 Table) for *Cryptosporidium* [43, 44] and *Giardia* [31], respectively. Both strands of the amplification products were sequenced using Big Dye Terminator version 3.1 reagents and an ABI 3730XL automated DNA sequencer (Applied Biosystems, Foster City, California, USA).

### Data analysis

#### DNA sequences analysis

Consensus sequences were assembled from forward and reverse reads and edited manually using Geneious v.10.1.3 [45]. The sequences derived were used to identify species and genotypes of *Cryptosporidium* and *Giardia* by aligning to sequence entries in nucleotide databases using the program BLAST (http://www.ncbi.nlm.nih.gov/blast/) and checked by their corresponding genotype [e.g., 46].

### Worldwide distribution of subtype families and assemblages

We extracted a dataset of available gp60 and *gdh* sequences from GenBank for the same species of *Cryptosporidium* and *Giardia* found in New Zealand. The search strategy used consist of one search string (species name) combined by the Boolean operator “AND” for gene name (gp60 or *gdh*). To check for duplicate sequences, accession numbers were compared between datasets from different countries. All results were verified and cleaned for further analyses (final searches were conducted on February 20, 2016). Sequences of gp60 and *gdh* for each specific country were allocated to the corresponding subtype families/assemblages following the source information from GenBank when available. In cases where this information was not available in GenBank we either aligned all sequences using SATé-II v.2.2.7 [47] or used BLAST software (http://www.ncbi.nlm.nih.gov/blast/) to determine the corresponding genotype. We complement this information with data from papers published in scientific journals (last search conducted on June 15, 2017) using the PubMed search engine via Geneious v.10.1.3. The search strategy for *Cryptosporidium* included the keywords gp60, gp40/15, gp15, pgp60, 60 kDa, 60 kilodalton glycoprotein, glycoprotein, genotype, subtype family, and cryptosporid*, whilst the search strategy for *Giardia* included the keywords gdh, glutamate dehydrogenase, assemblage, and giard* [30]. Geographical distributions were visualized as maps using ggplot2 [48] and rworldmap [49] packages in RStudio v.0.98.1091.

### Extrapolation and similarity of parasite genotypes

We used a proportional similarity index (PSI) in the R package spaa [50] to calculate niche overlap (co-occurrence) patterns among countries according to the type and number of genetic variants reported. Values close to 1 indicate very similar countries in their genotype diversity, whereas values close to 0 indicate countries with dissimilar genotypes. Next, we used the “specaccum” function in the R package vegan [51] to generate genotypes (cf. species) accumulation curves [52] by plotting the total number of different subtype families/assemblages against the number of all countries reporting data on GenBank combined and all countries in the world (~200). Rarefaction was used to produce smooth mean parasite genotypes variability and calculate 95% upper and lower bounds.

Pairwise *F*_ST_ and an analysis of molecular variance (AMOVA) were performed in Arlequin v.3.5.2.2 [53] using the sequence data obtained from GenBank to recognize the genetic differentiation between pairs of populations and the level of population genetic structure, respectively. Gblocks v.0.91b [54] was used to remove nonconserved positions adjacent to gaps and nucleotide repeats in the protein coding-gene gp60.

### Accession numbers

DNA sequences have been submitted to GenBank: accession numbers KY123918–KY124121. Note that only unique haplotypes were submitted but similar haplotypes found in different hosts were repeatedly submitted (see S2 Table).

## Results

### Molecular identification of species and genotypes

**Fig 2:**
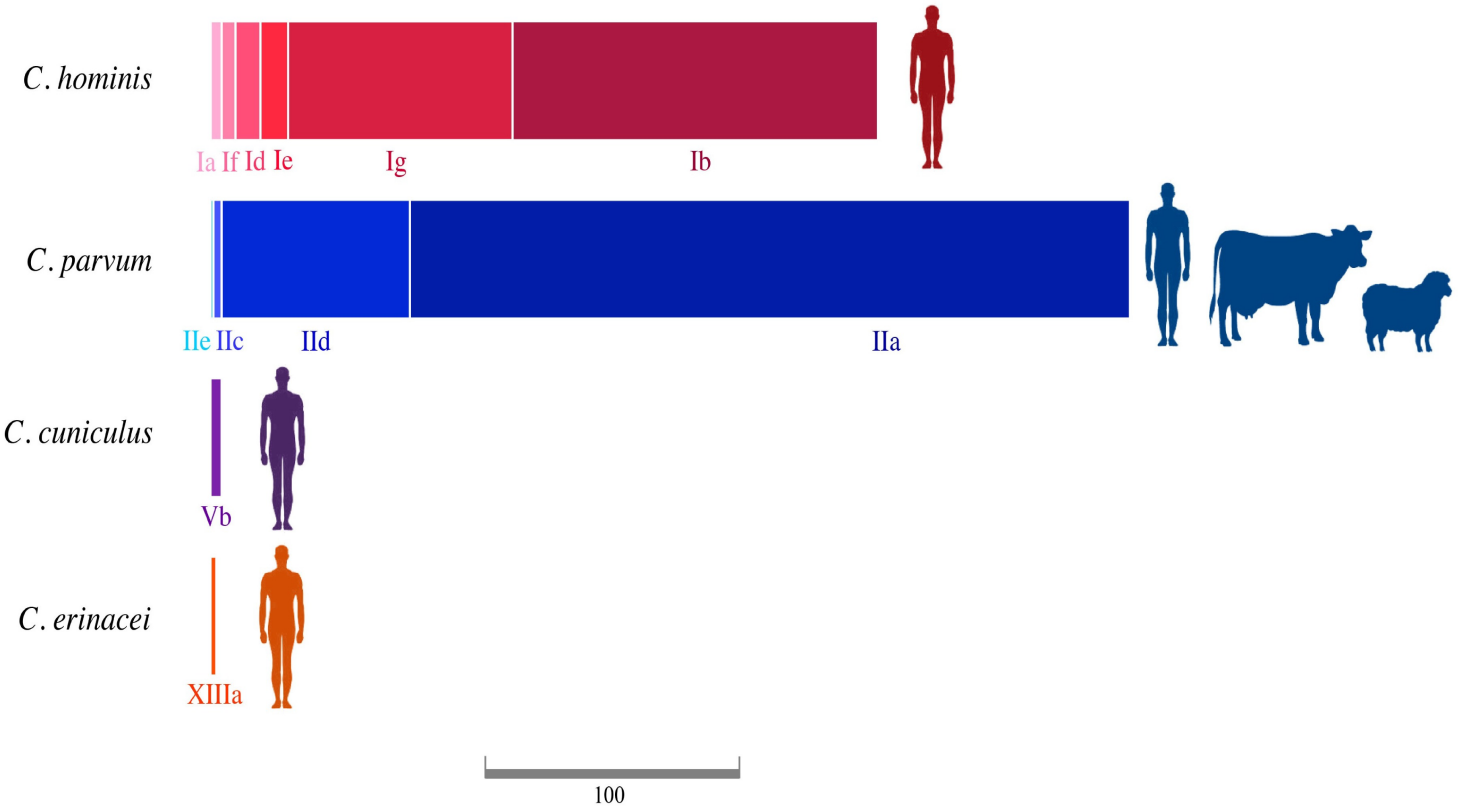
Subtype families and hosts of *Cryptosporidium* species found in this study. The size of the squares is proportional to the number of samples identified for each subtype family. The scale bar corresponds to 100 samples. See S3 Table for details.

**Fig 3:**
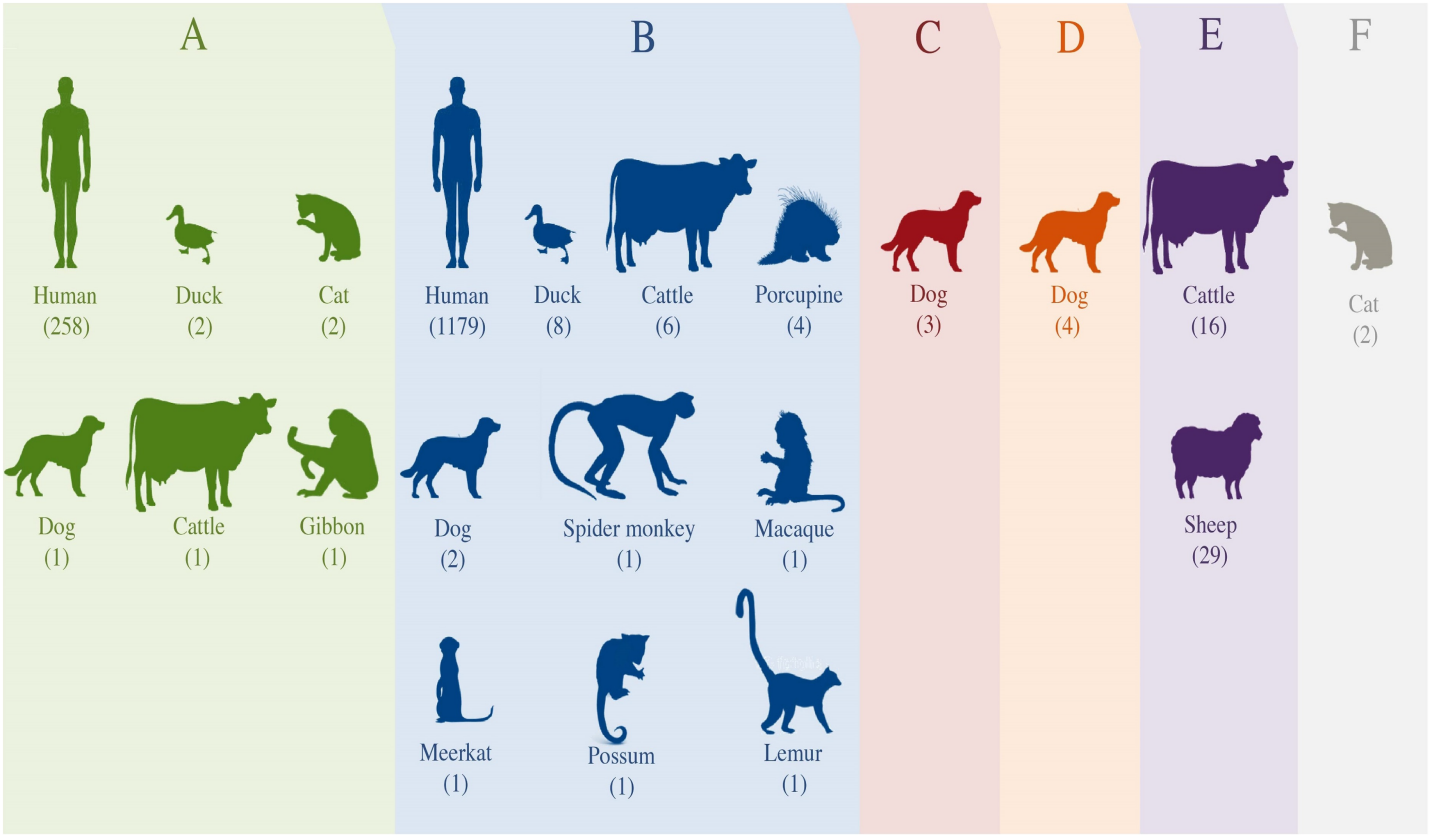
Range of hosts and assemblages (A to F) of *Giardia intestinalis* found in New Zealand. The number of samples by hosts identified within each assemblage is found in brackets.

A total of 579 sequences were obtained for *Cryptosporidium* and 1523 for *Giardia* (S3 Table). The sequences presented > 98% identity and E-value of 0.0 with BLAST searching against sequences in GenBank, confirming a reliable identification of species and genotypes. *Cryptosporidium* species detected in New Zealand using the gp60 marker were *C*. *parvum*, *C*. *hominis*, *C*. *cuniculus* and *C*. *erinacei*. Our molecular analysis identified 332 samples with the presence of *C*. *parvum*, 241 *C*. *hominis*, four *C*. *cuniculus* and two *C*. *erinacei*. Samples of *C*. *parvum* were collected from humans (86.4%), cattle (13%) and sheep (0.6%), whilst the remaining species were collected only from humans (Fig 2 and S3 Table). Subtype families found in *C*. *parvum* were IIa, IIc, IId and IIe (Fig 2). The most common subtype families were IIa (78.3%) and IId (20.5%). The subtype families in *C*. *hominis* most frequently identified were Ib (54.8%) and Ig (33.6%). Other subtype families found in our study were Ia, Id, Ie and If, which represented the remaining 11.6% (Fig 2 and S3 Table). *Cryptosporidium cuniculus* and *C*. *erinacei* were assigned to subfamilies Vb and XIIIa, respectively (Fig 2 and S3 Table). Assemblages from A to F of *G*. *intestinalis* are presented in New Zealand but assemblage B is notably dominant (79%) and found in humans, domestic, wild and zoo animals (Fig 3 and S3 Table).

### Global geographic distribution of gp60 subtype families and *gdh* assemblages

**Fig 4:**
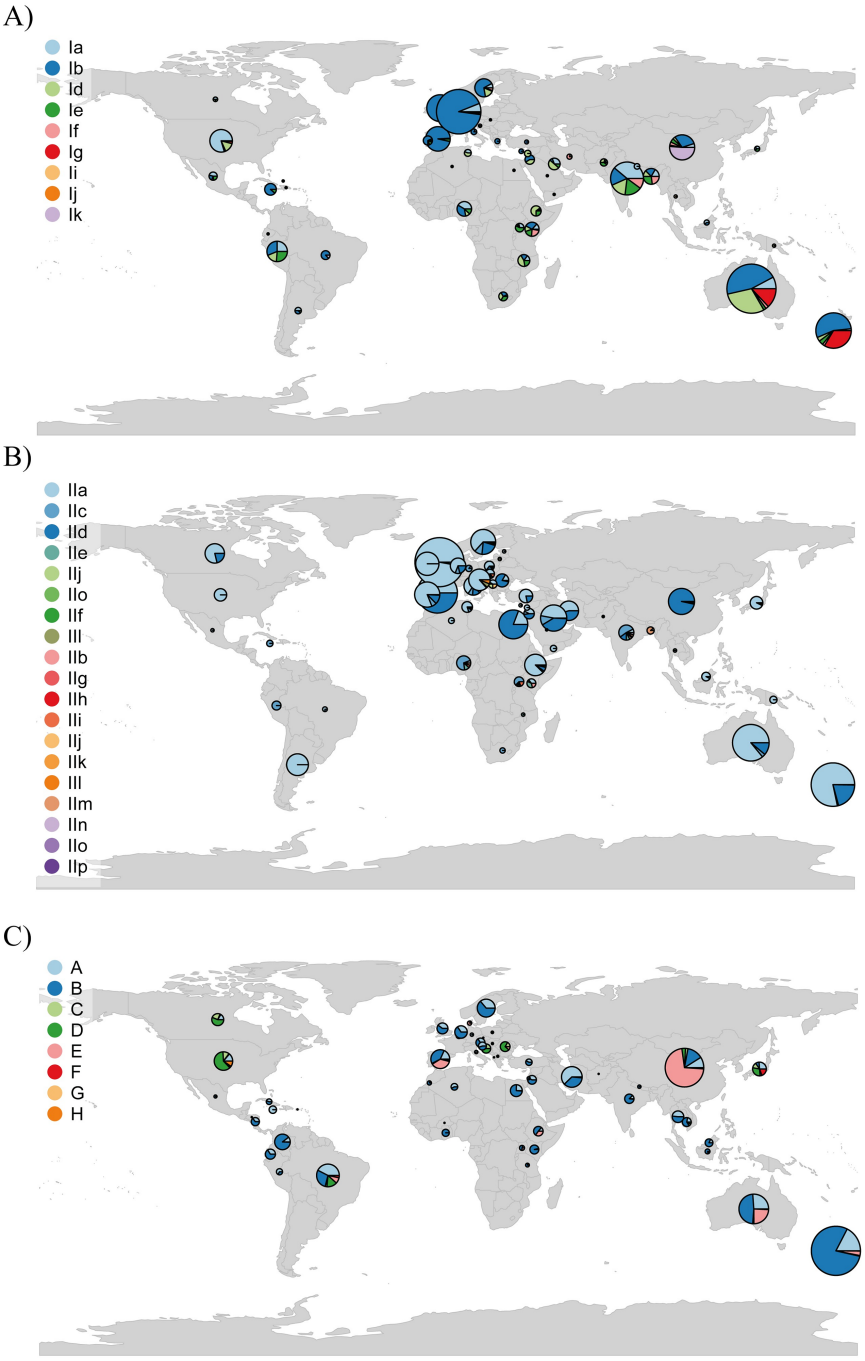
Global records of *Cryptosporidium* and *Giardia* genotypes data. (a) and (b) Distribution of gp60 subtype families in *C*. *hominis* and *C*. *parvum*, respectively. (c) Distribution of *gdh* assemblages in *Giardia intestinalis*. The size of the pies is proportional to the frequency of data obtained from publications and GenBank data. Data for New Zealand includes this study.

*Cryptosporidium parvum* and *C*. *hominis* were found across all continents (Fig 4a and 4b and S4 Table) but Australia and the United Kingdom accounted for the majority of data for both pathogens (S4 Table). Ten subtype families of *C*. *hominis* (Ia-Ik) are found around the world whilst 16 have been identified for *C*. *parvum* (IIa-IIp). *Cryptosporidium hominis* subtype families Ia, Ib, Id and Ie are the most abundant worldwide (S4 Table). Regardless of the high diversity of subtype families in *C*. *parvum*, IIa and IId are the most abundant. A more restricted geographical distribution is reported for *C*. *cuniculus* and *C*. *erinacei*, with data from Australia, China, Czech Republic, Poland, the United Kingdom, Algeria and Germany (S4 Table). Both of the current recognized genotypes for *C*. *cuniculus* (Va and Vb) were reported in China and the United Kingdom but Vb has been additionally found in Australia, Czech Republic and Poland.

We extracted 4348 records corresponding to *G*. *intestinalis* assemblages from 64 countries spanning all continents except Antarctica (Fig 4c and S4 Table). However, the majority of available data were retrieved from China, Australia and Brazil. Assemblages A and B represent more than 60% of the reported data worldwide (S4 Table). There are two additional assemblages (G and H) that are not found in New Zealand. Assemblage G is reported in Australia, China and Spain whilst assemblage H is found in Colombia and USA.

### Countries similarity and global genotypes accumulation curves

**Fig 5:**
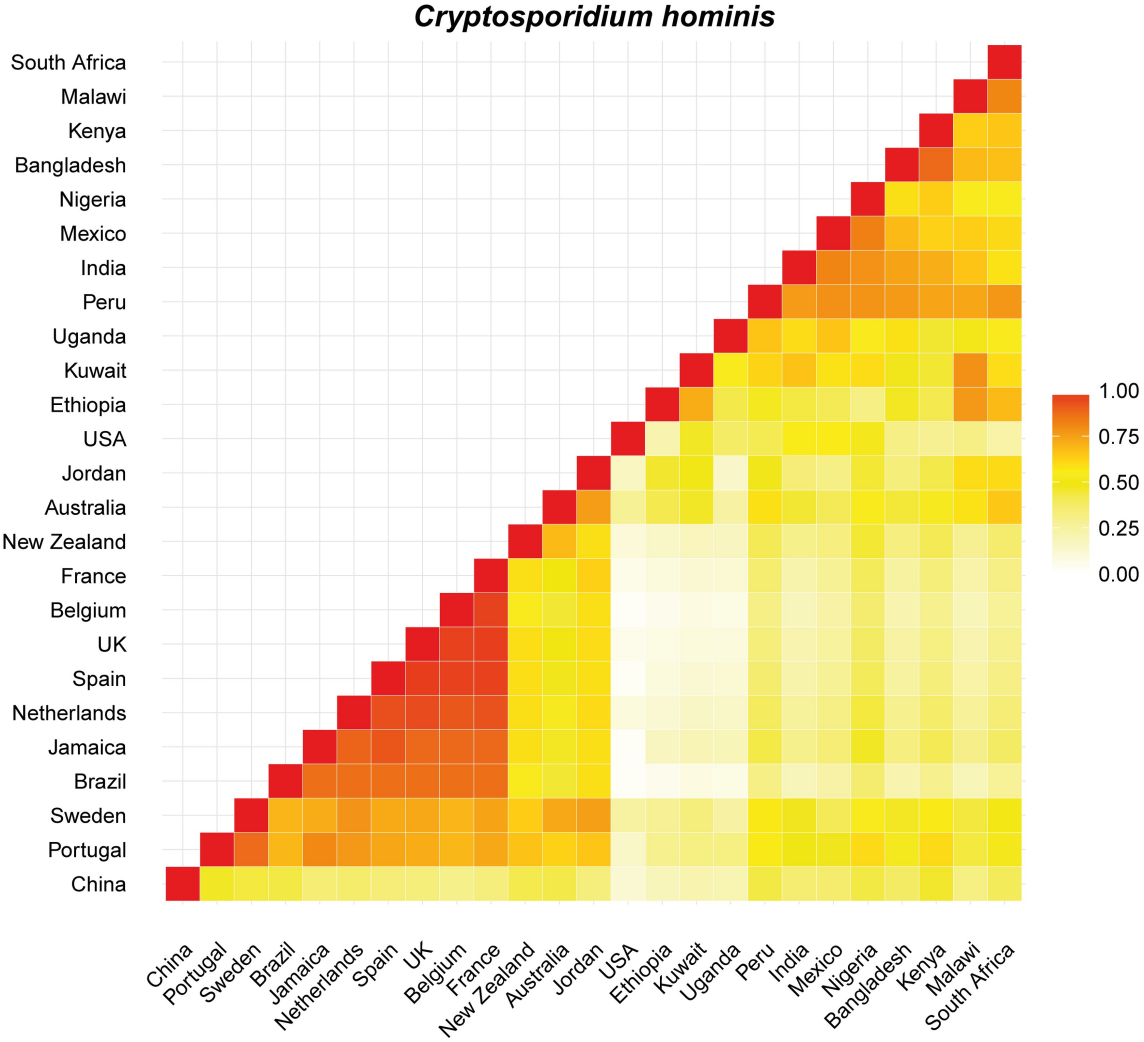
Plot showing the proportional similarity of *C*. *hominis* genotypes between pairs of countries. Highly similar countries are showed in darker colours.

**Fig 6:**
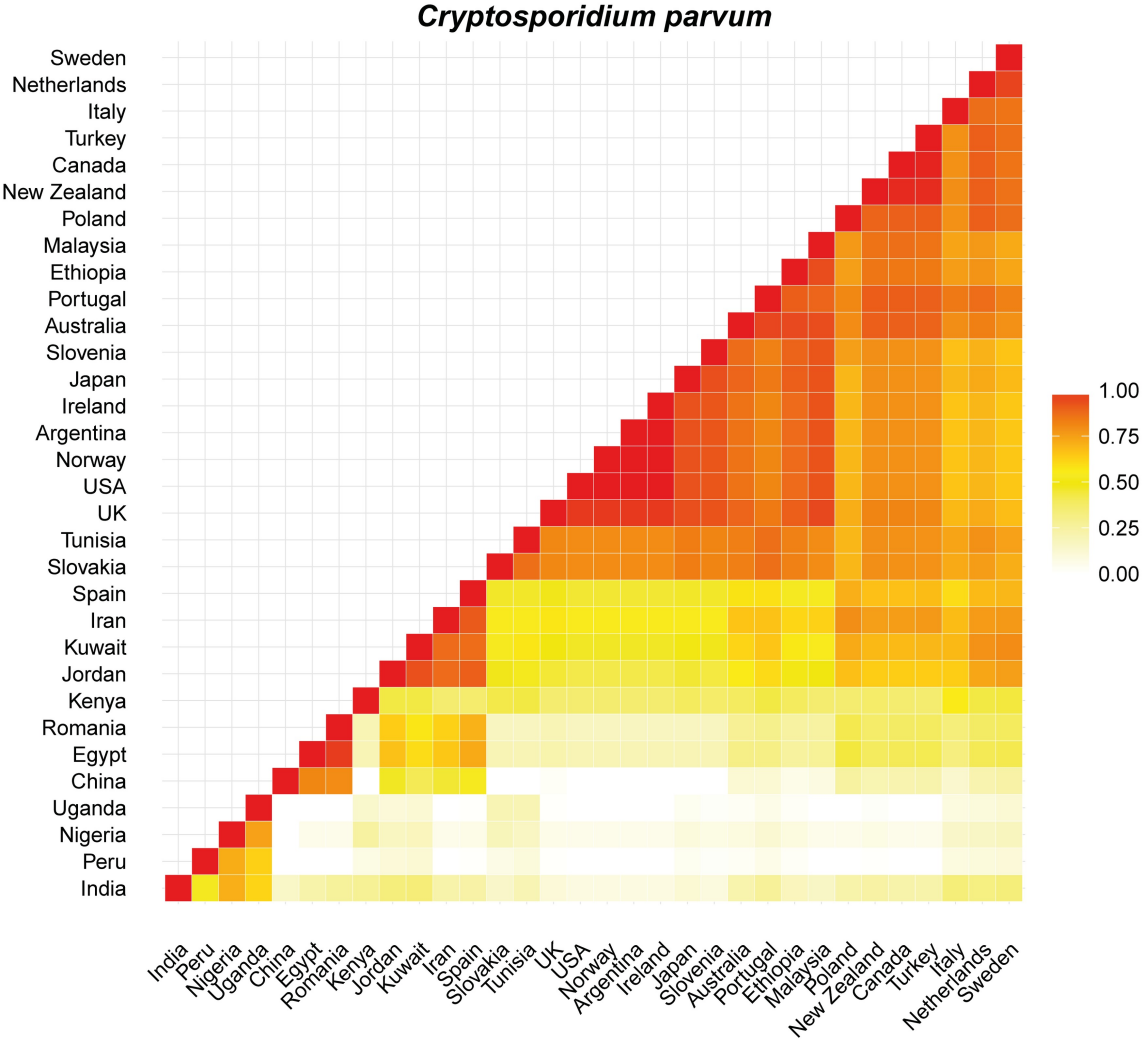
Plot showing the proportional similarity of *C*. *parvum* genotypes between pairs of countries. Highly similar countries are showed in darker colours.

**Fig 7:**
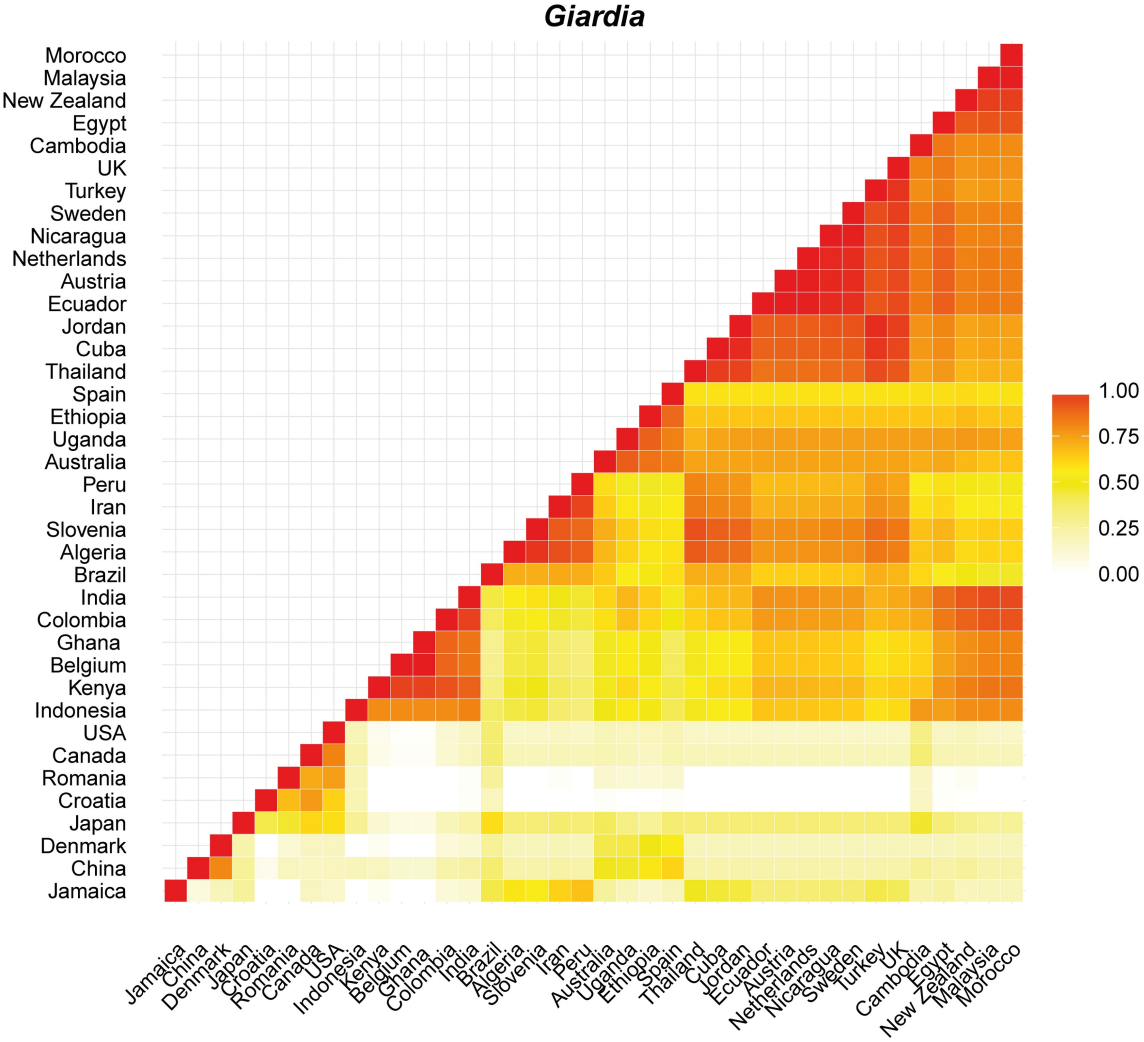
Plot showing the proportional similarity of *G*. *intestinalis* genotypes between pairs of countries. Highly similar countries are showed in darker colours.

Proportional similarity analyses were carried out only between countries with more than 10 records retrieved. In general, *C*. *hominis* showed intermediate to high values (> 0.5) of similarity for most country comparisons with New Zealand visually similar to Australia, Portugal, Sweden and the United Kingdom (Figs 5 and S1). *Cryptosporidium parvum* showed high similarity (> 0.75) among most country comparisons with exception of comparisons against Uganda, Nigeria, China, Peru and India (Figs 6 and S2). In this case, New Zealand is most like countries from Europe, including the Netherlands, Sweden, the United Kingdom and Portugal and countries from other hemispheres such as Canada and Australia. High values of similarity were also observed for comparisons of *G*. *intestinalis* between countries spanning different continents but Denmark, Croatia, Romania, Canada and USA were visually dissimilar (< 0.5) (Figs 7 and S3). New Zealand is similar to many countries from across the world, from Europe to Asia, Africa and Australia (Fig 7).

**Fig 8:**
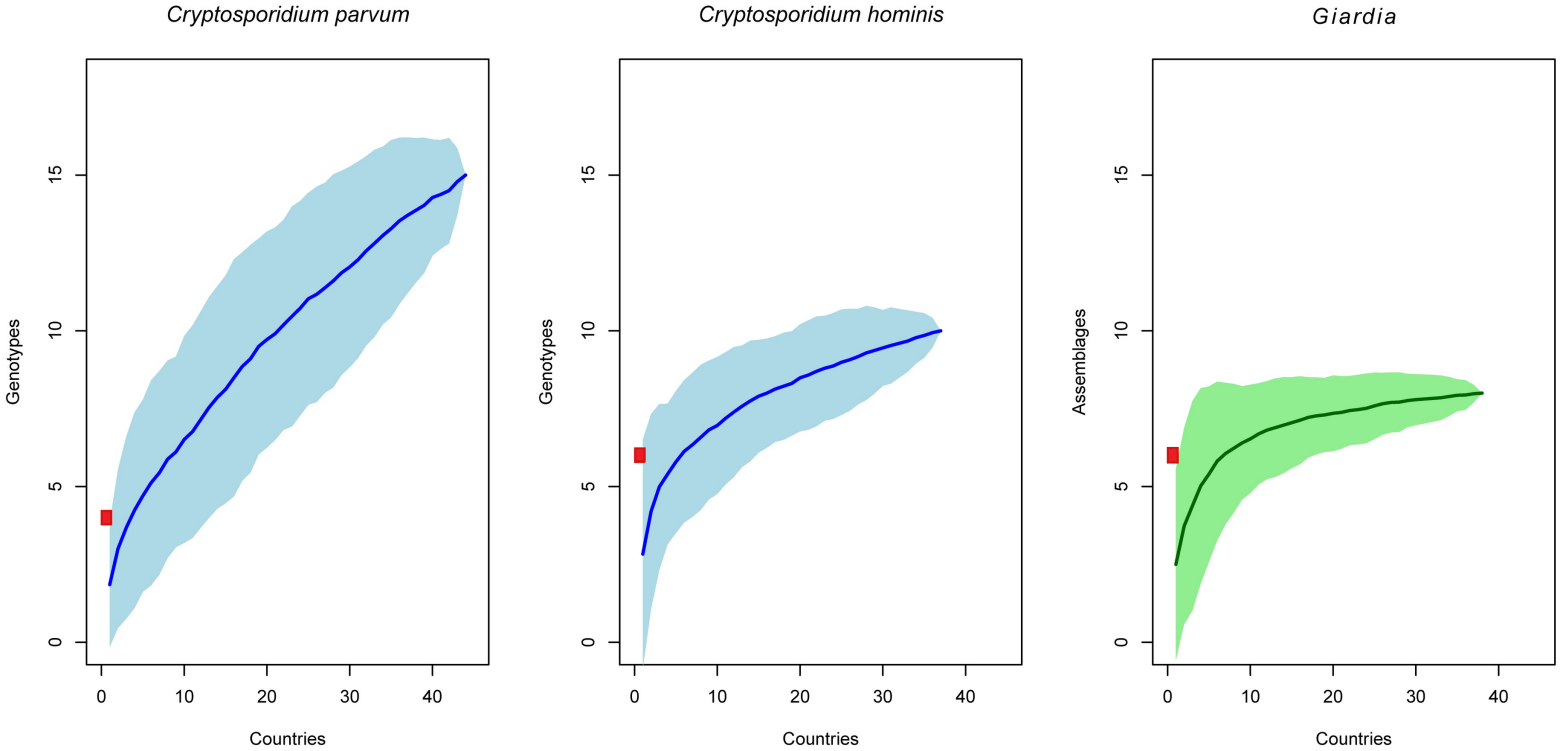
Genotype accumulation curves of *C*. *parvum* (left), *C*. *hominis* (center) and *G*. *intestinalis* (right) estimated by the number of countries with reported data. For each curve the lighter shaded region shows 95% confidence intervals and the red square correspond to the number of genotypes for each species found in New Zealand.

Fig 8 shows the cumulative number of genotypes for all countries combined. Accumulation curves for *G*. *intestinalis* and *C*. *hominis* were starting to show some downward curvature below 10 genotypes, which indicates a declining rate of parasite genetic type discovery, but only *G*. *intestinalis* approached an asymptote. The number of genetic types found in New Zealand for these two species is close to the plateau area of the curves (Fig 8). Interestingly, *C*. *parvum* show a different pattern with an upward curvature which indicates that many more genetic types than those currently recorded will be found if sampling in other countries is performed. However, this species may never attain an asymptote (Figs 8 and S4) indicating that other genotypes will be likely detected worldwide as well as in New Zealand. Rarefaction analysis using only countries with more than 10 records did not show differences of these trends (S5 Fig).

Population structure analyses were carried out between countries with more than 10 sequences available in GenBank. Sequences that do not overlap at least 50% of the total length of the dataset were discarded. Gaps and nucleotide repeats were not considered for pairwise *F*_ST_ and AMOVA analyses. Large and significant *F*_ST_ values are an indication of population subdivision and/or low gene flow between populations. There was not significant (*p* < 0.0001) population differentiation of comparisons among Nigeria, Mexico, Kenya and USA for *C*. *hominis*. New Zealand showed high significant pairwise *F*_ST_ when compared with other countries with exception of comparisons among Nigeria, Mexico, USA and Australia (S5 Table). A lack of population differentiation for *C*. *parvum* was evident between most comparisons of estimated pairwise *F*_ST_ (S5 Table). We did not find structured populations among closely geographical European countries (e.g. United Kingdom, Sweden, Spain, Slovenia and Poland) as well as other countries spanning around the world, with exception of most comparisons among Egypt, India and China (S5 Table). New Zealand showed no population differentiation with Australia, Argentina, USA, Sweden, Poland, Slovenia and Turkey. *Giardia intestinalis* also revealed no population subdivision among most countries including those from different continents, with exception of most comparisons among Ethiopia, Egypt, Denmark, Croatia, Romania and Colombia. New Zealand presented no population subdivision with countries from different latitudes including Norway, Malaysia, Uganda, Sweden, Indonesia, Japan, Germany, Australia, among others (S5 Table). However, there was substantial polymorphism within populations (> 80%) found in the AMOVA which result in low but significant proportions of the total variation partitioned among countries (14%, 19% and 16%, *p* < 0.0001 for *C*. *hominis*, *C*. *parvum* and *G*. *intestinalis*, respectively).

## Discussion

Our relatively large sample size and genotyping diversity of *Cryptosporidium* and *Giardia* from New Zealand allowed us to perform comparative analyses with available worldwide data. All species and genetic variants reported in New Zealand of either *Cryptosporidium* or *Giardia* are found in several countries and this homogenization suggests that their populations are well established and mixed throughout the globe [55, 56]. Some cases of structure relating to geography have been indicated by previous studies [e.g., 57, 58] but they also show sharing alleles among different populations around the world. We also found evidence of geographically-associated differentiation with high *F*_ST_ values that were generally confirmed by low PSI values, despite large confidence intervals (S1, S2 and S3 Figs). The evidence of structure is likely a consequence of limitations in the estimators due to an uneven number of samples, high within-population variation and reporting bias [59]. The use of a single and high variable locus and non-contemporaneous samples also affect the probability of a reliable estimation of the genetic differentiation from sequence data [60, 61]. Furthermore, the population structure of these species, going from clonal to panmictic, is influenced by diverse factors such as transmission dynamics and host-specificity [57, 62].

The global distribution of species and genotypes within *Cryptosporidium* and *Giardia* might represent the geographic range shifts of domesticated animals, historical movement of livestock and transmission facilitated by human travel and non-native fauna [35, 63, 64]. This is likely the case of the rare *C*. *cuniculus* and *C*. *erinacei* causing human disease in New Zealand. *Cryptosporidium cuniculus* has recently expanded its known host range from rabbits to humans and kangaroos [65] and it is a human pathogen of public health importance related to waterborne outbreaks in the United Kingdom [66]. The presence of this species and a similar genotype in New Zealand and few other countries may suggest a dispersal event caused by human factors. Most recently, *C*. *erinacei* has been identified as a cause of gastroenteritis in immunocompetent man in the Czech Republic, having previously only been identified in hedgehogs and horses [20]. Though we have no data on the origin of the infection, finding that humans in New Zealand have been infected with *C*. *erinacei* could be an indication of cross-species transmission events from either domestic or wildlife hosts, introduced pets or non-native mammals such as possums, hedgehogs, rabbits and horses. Specific future studies could include active surveillance of human, domestic and wild animal populations and longitudinal studies with advanced molecular methods [16]. More systematic sampling of local and introduced fauna along with whole genome sequences over short timescales will allow us to identify major functional loci involved in host specificity, proliferation and host shifting. This information will provide insights into the zoonotic potential of different genotypes, host range, cross-species transmission and risks for host shifts to humans [67].

We acknowledge that *Cryptosporidium* species diversity will be underestimated because most species within the genus fail to amplify with standard primers used for the gp60 marker [68, 69]. For instance, *C*. *andersoni* and *C*. *bovis* have been previously identified in cows in New Zealand using the 18S rRNA gene markers [70]. Moreover, our approaches are dependent on sequences submission, accurate taxonomy and complementary information in GenBank. Some genotypes have been recognized as species or wrongly submitted to a different species. *Cryptosporidium erinacei* was previously described as *C*. sp. hedgehog [20] with the single XIIIa subtype family but a subtype family VIIa was also proposed (S4 Table) [71]. Likewise, the subtype family IIf in *C*. *parvum* has recently been classified as *C*. *tyzzeri* [72]. This is also the case for *G*. *intestinalis*. Several assemblages of *G*. *intestinalis* are genetically isolated lineages and have been nominated as different species [73]. Some genotypes in *C*. *parvum* and *G*. *intestinalis* might be named as new species when more data become available.

Sequences from public databases are an extremely valuable source of information [74]. Genotypes and abundance in different countries may not be totally represented because some researchers avoid submitting multiple sequences if the same genotype is already found within the sequence data collection of GenBank [74]. The potential bias caused by this practice does not help to capture a substantial part of the genetic diversity globally. This information is fundamental for analyses of the evolution and movement of the parasites, the possible origin and patterns of dispersal of these organisms or their genotypes, prioritization on potential hazard reduction to susceptible hosts and control to prevent spread between regions. For example, *C*. *parvum* haplotypes IIg, IIh, IIi are reported in Uganda, IIm in Bangladesh, and IIn in India; *C*. *hominis* Ii is found in Asia; and *G*. *intestinalis* assemblage G in Australia. Given current reporting practices, it is unclear if these genotypes are unique to these regions deserving greater attention or if they are widely distributed but simply unreported on GenBank. Using our literature review framework (S1 References), we found that subtype families IIg, IIi and IIm were also reported in India, Ii in USA and assemblage G in China and Spain. We predict that unreported sequences from different countries will decrease the geographical population structure, however, it will be dependent on whether researchers do not report sequences because of 100% identity or because they are the same genotype. Another alternative source of information to measure the population genetic variation of these parasites is ZoopNet [75, 76] but this database is not publicly available. A previous study using ZoopNet found high genetic diversity in *C*. *hominis* and *C*. *parvum* from Australia and the United Kingdom, respectively [75]. Estimations of the genetic diversity in this study align with our results and the similarity between these nations and New Zealand.

It has been demonstrated that mixed infections (different alleles per isolate) within a host can produce recombinant genotypes that comprise alleles inherited from each parental line [77, 78]. These recombinant events are associated to the high probability of finding new genotypes (S4 Fig). *Cryptosporidium parvum* displayed a large genotypic variation as a result of gene combinations and host range shift [67]. Similarly, genetic recombination has been shown to occur commonly in *C*. *hominis* and *G*. *intestinalis* in developing countries [59, 78]. This is an important issue for public health risks because genetic recombination is a driving force for the emergence of virulent subtypes [79]. The distribution of these species in humans might vary among geographical areas and socioeconomic conditions [68] and interestingly, high genetic diversity and new genotypes have been particularly found in children or unexpected hosts in rural settings from developing countries [e.g., 80, 81–85]. Unfortunately, the data reported from developing countries is low with limited information from Africa, Latin America and populous countries such as China and India [30].

Comprehensive surveillance strategies will require efforts at multiple scales to help decrease the significant burden caused by *Cryptosporidium* and *Giardia*. Understanding of the transmission pathways of these parasitic protozoans is imperative. The large number of zoonotic cases highlights the importance of rates of contact and host densities. For example, local spread of *Cryptosporidium* and *Giardia* in New Zealand has been facilitated by cattle densities in the catchment of drinking water particularly within the most populous and agricultural regions [37, 39, 42, 86]. It is uncertain in our study if this is the transmission pathway, particularly for *Giardia* which shows less seasonality in case notification rates (Fig 1), but a substantial number of diarrheal cases around the world suggest that infections among humans has an increasing trend caused by waterborne transmission [e.g., 24, 87]. Animal exposure, seasonal patterns and contaminated food are other key factors for transmission in certain geographical settings and more detailed analysis will be need to identify the pathways of human cryptosporidiosis and giardiasis.

## Supporting information

S1 Fig Plot showing the proportional similarity of *C*. *hominis* genotypes between pairs of countries. Highly similar countries are showed in darker colours and 95% lower (above) and upper (below) bootstrapped confidence limits in numbers within each box.
(TIF)

S2 Fig Plot showing the proportional similarity of *C*. *parvum* genotypes between pairs of countries. Highly similar countries are showed in darker colours and 95% lower (above) and upper (below) bootstrapped confidence limits in numbers within each box.
(TIF)

S3 Fig Plot showing the proportional similarity of *G*. *intestinalis* genotypes between pairs of countries. Highly similar countries are showed in darker colours and 95% lower (above) and upper (below) bootstrapped confidence limits in numbers within each box.
(TIF)

S4 Fig One hundred generations of genotype accumulation curves of *C*.*parvum* (left), *C*. *hominis* (center) and *G*. *intestinalis* (right) by sampling all countries in the world (~200). (TIF)

S5 Fig Genotype accumulation curves of *C*. *parvum* (left), *C*. *hominis* (center) and *G*. *intestinalis* (right) estimated by the number of countries with more than 10 records. For each curve the lighter shaded region shows 95% confidence intervals and the red square correspond to the number of genotypes for each species found in New Zealand.
(TIF)

S1 Table Primers for PCR and DNA sequencing employed in this study. (DOCX)

S2 Table Species, sample ID, haplotype number, host, genotypes, number of sequences per haplotype and GenBank accession numbers of data included in this study. (DOCX)

S3 Table Taxa, genotypes, number of sequences, percentage of occurrence and range of hosts of *Cryptosporidium* and *Giardia* species found in this study. (DOCX)

S4 Table Taxa, country, frequency of subtype families/assemblages and list of references. Details for the literature used on this data are found in the accompanying pdf document.
(XLSX)

S5 Table *F*_ST_ between pairs of populations (countries) of *C*. *hominis*, *C*. *parvum* and *G*. *intestinalis* using sequence data from the gp60 and *gdh* genes. Highly significant values (*p* < 0.0001) are shown in red.
(XLSX)

S1 References References for S4 Table. (PDF)

## References

[1] Morens DM, Folkers GK, Fauci AS. The challenge of emerging and re-emerging infectious diseases. Nature. 2004;430(6996):242–9. 10.1038/nature02759 15241422 PMC7094993

[2] Morse SS. Factors in the emergence of infectious diseases. Emerging Infectious Diseases. 1995;1(1):7–15. 10.3201/eid0101.950102 8903148 PMC2626828

[3] Currie J, Grenfell B, Farrar J. Beyond Ebola. Science. 2016;351(6275):815–6. 10.1126/science.aad8521 26912880 PMC5134423

[4] Espinal M, Aldighieri S, John RS, Becerra-Posada F, Etienne C. International health regulations, Ebola, and emerging infectious diseases in Latin America and the Caribbean. American Journal of Public Health. 2015;106(2):279–82. 10.2105/AJPH.2015.302969 26691130 PMC4815620

[5] Woolhouse MEJ, Rambaut A, Kellam P. Lessons from Ebola: Improving infectious disease surveillance to inform outbreak management. Science Translational Medicine. 2015;7(307):1–9. 10.1126/scitranslmed.aab0191 PMC5819730 26424572

[6] Heymann D, L., Rodier G. Global Surveillance, National Surveillance, and SARS. Emerging Infectious Diseases. 2004;10(2):173–5. 10.3201/eid1002.031038 15040346 PMC3322938

[7] Wilson ME. Travel and the emergence of infectious diseases. Emerging Infectious Diseases. 1995;1(2):39–46. 10.3201/eid0102.952001 8903157 PMC2626831

[8] Weiss RA, McMichael AJ. Social and environmental risk factors in the emergence of infectious diseases. Nature Medicine. 2004;10(12):S70—S6. 10.1038/nm1150 PMC7095886 15577934

[9] Jones KE, Patel NG, Levy MA, Storeygard A, Balk D, Gittleman JL, et al Global trends in emerging infectious diseases. Nature. 2008;451(7181):990–3. http://www.nature.com/nature/journal/v451/n7181/suppinfo/nature06536_S1.html. 10.1038/nature06536 18288193 PMC5960580

[10] Mead PS, Slutsker L, Dietz V, McCaig LF, Bresee JS, Shapiro C, et al Food-related illness and death in the United States. Emerging Infectious Diseases. 1999;5(5):607–25. 10.3201/eid0505.990502 10511517 PMC2627714

[11] Enserink R, van den Wijngaard C, Bruijning-Verhagen P, van Asten L, Mughini-Gras L, Duizer E, et al Gastroenteritis attributable to 16 enteropathogens in children attending day care: Significant effects of Rotavirus, Norovirus, Astrovirus, Cryptosporidium and Giardia. The Pediatric Infectious Disease Journal. 2015;34(1):5–10. 10.1097/INF.0000000000000472 24983718

[12] Ng-Hublin JSY, Hargrave D, Combs B, Ryan U. Investigation of a swimming pool-associated cryptosporidiosis outbreak in the Kimberley region of Western Australia. Epidemiology & Infection. 2015;143(05):1037–41. 10.1017/S095026881400106X 25703474 PMC9507107

[13] Gertler M, Dürr M, Renner P, Poppert S, Askar M, Breidenbach J, et al Outbreak of cryptosporidium hominis following river flooding in the city of Halle (Saale), Germany, August 2013. BMC Infectious Diseases. 2015;15(1):1–10. 10.1186/s12879-015-0807-1 25879490 PMC4344771

[14] Savioli L, Smith H, Thompson A. *Giardia* and *Cryptosporidium* join the ‘Neglected Diseases Initiative’. Trends in Parasitology. 2006;22(5):203–8. 10.1016/j.pt.2006.02.015. 16545611

[15] Cacciò SM, Thompson RCA, McLauchlin J, Smith HV. Unravelling *Cryptosporidium* and *Giardia* epidemiology. Trends in Parasitology. 2005;21(9):430–7. 10.1016/j.pt.2005.06.013. 16046184

[16] Checkley W, White AC Jr, Jaganath D, Arrowood MJ, Chalmers RM, Chen X-M, et al A review of the global burden, novel diagnostics, therapeutics, and vaccine targets for cryptosporidium. The Lancet Infectious Diseases. 2015;15(1):85–94. 10.1016/S1473-3099(14)70772-8. 25278220 PMC4401121

[17] Rossignol J-F. Cryptosporidium and Giardia: Treatment options and prospects for new drugs. Experimental Parasitology. 2010;124(1):45–53. 10.1016/j.exppara.2009.07.005. 19632225

[18] Broglia A, Weitzel T, Harms G, Cacció SM, Nöckler K. Molecular typing of *Giardia duodenalis* isolates from German travellers. Parasitol Res. 2013;112(10):3449–56. 10.1007/s00436-013-3524-y 23892479

[19] Sallon S, Deckelbaum RJ, Schmid II, Harlap S, Baras M, Spira DT. *Cryptosporidium*, malnutrition, and chronic diarrhea in children. American Journal of Diseases of Children. 1988;142(3):312–5. 10.1001/archpedi.1988.02150030086027 3344720

[20] Kváč M, Saková K, Kvĕtoňová D, Kicia M, Wesołowska M, McEvoy J, et al Gastroenteritis caused by the *Cryptosporidium* hedgehog genotype in an immunocompetent man. Journal of Clinical Microbiology. 2014;52(1):347–9. 10.1128/JCM.02456-13 24131692 PMC3911458

[21] Kotloff KL, Nataro JP, Blackwelder WC, Nasrin D, Farag TH, Panchalingam S, et al Burden and aetiology of diarrhoeal disease in infants and young children in developing countries (the Global Enteric Multicenter Study, GEMS): a prospective, case-control study. The Lancet. 2013;382(9888):209–22. 10.1016/S0140-6736(13)60844-2 23680352

[22] Lima A, Samie A, Guerrant R. Cryptosporidiosis In: Guerrant R, Walker D, Weller P, editors. Tropical Infectious Diseases. Philadelphia, USA: Elsevier-Churchill Livingstone; 2011 p. 640–63.

[23] Feng Y, Xiao L. Zoonotic potential and molecular epidemiology of *Giardia* species and giardiasis. Clinical Microbiology Reviews. 2011;24(1):110–40. 10.1128/CMR.00033-10 21233509 PMC3021202

[24] Baldursson S, Karanis P. Waterborne transmission of protozoan parasites: Review of worldwide outbreaks—An update 2004–2010. Water Research. 2011;45(20):6603–14. 10.1016/j.watres.2011.10.013. 22048017

[25] Lane S, Lloyd D. Current trends in research into the waterborne parasite *Giardia*. Critical Reviews in Microbiology. 2002;28(2):123–47. 10.1080/1040-840291046713 12109771

[26] WHO. Fighting Disease Fostering Development. Geneva: 1996.

[27] Xiao L, Escalante L, Yang C, Sulaiman I, Escalante AA, Montali RJ, et al Phylogenetic analysis of *Cryptosporidium* parasites based on the small-subunit rRNA gene locus. Applied and Environmental Microbiology. 1999;65(4):1578–83. 10103253 10.1128/aem.65.4.1578-1583.1999 PMC91223

[28] Fayer R. Taxonomy and species delimitation in *Cryptosporidium*. Experimental Parasitology. 2010;124(1):90–7. 10.1016/j.exppara.2009.03.005. 19303009

[29] McDonnell PA, Upcroft J, Upcroft P, Buret A. Morphological identification markers for distinguishing avian from mammalian *Giardia* species—do they need reconsideration? European Journal of Protistology. 2001;37(3):273–80. 10.1078/0932-4739-00818.

[30] Jex AR, Gasser RB. Genetic richness and diversity in *Cryptosporidium hominis* and *C*. *parvum* reveals major knowledge gaps and a need for the application of “next generation” technologies—Research review. Biotechnology Advances. 2010;28(1):17–26. 10.1016/j.biotechadv.2009.08.003. 19699288

[31] Read CM, Monis PT, Andrew Thompson RC. Discrimination of all genotypes of *Giardia duodenalis* at the glutamate dehydrogenase locus using PCR-RFLP. Infection, Genetics and Evolution. 2004;4(2):125–30. 10.1016/j.meegid.2004.02.001. 15157630

[32] Jex AR, Smith HV, Monis PT, Campbell BE, Gasser RB. Cryptosporidium—Biotechnological advances in the detection, diagnosis and analysis of genetic variation. Biotechnology Advances. 2008;26(4):304–17. 10.1016/j.biotechadv.2008.02.003. 18430539

[33] Strong WB, Gut J, Nelson RG. Cloning and sequence analysis of a highly polymorphic *Cryptosporidium parvum* gene encoding a 60-kilodalton glycoprotein and characterization of its 15- and 45-kilodalton zoite surface antigen products. Infection and Immunity. 2000;68(7):4117–34. 10858229 10.1128/iai.68.7.4117-4134.2000 PMC101708

[34] Monis PT, Mayrhofer G, Andrews RH, Homan WL, Limper L, Ey PL. Molecular genetic analysis of Giardia intestinalis isolates at the glutamate dehydrogenase locus. Parasitology. 1996;112(01):1–12. 10.1017/S0031182000065021 8587793

[35] Wang R, Zhang L, Axen C, Bjorkman C, Jian F, Amer S, et al *Cryptosporidium parvum* IId family: clonal population and dispersal from Western Asia to other geographical regions. Scientific Reports. 2014;4(4208):srep04208. 10.1038/srep04208 PMC3936226 24572610

[36] Putignani L, Menichella D. Global distribution, public health and clinical impact of the Protozoan pathogen *Cryptosporidium*. Interdisciplinary Perspectives on Infectious Diseases. 2010;2010:39 10.1155/2010/753512 20706669 PMC2913630

[37] Snel SJ, Baker MG, Kamalesh V, French N, Learmonth J. A tale of two parasites: the comparative epidemiology of cryptosporidiosis and giardiasis. Epidemiology & Infection. 2009;137(11):1641–50. 10.1017/S0950268809002465 19393124

[38] Lal A, Ikeda T, French N, Baker MG, Hales S. Climate variability, weather and enteric disease incidence in New Zealand: Time series analysis. PLoS ONE. 2013;8(12):e83484 10.1371/journal.pone.0083484 24376707 PMC3871872

[39] Learmonth JIM, Ionas G, Pita A, Cowie R. Seasonal shift in *Cryptosporidium parvum t*ransmission cycles in New Zealand. Journal of Eukaryotic Microbiology. 2001;48:34–5. 10.1111/j.1550-7408.2001.tb00444.x 11906069

[40] ESR. Surveillance report: Notifiable diseases in New Zealand Annual report 2014. Porirua, New Zealand: 2015.

[41] Binney BM, Biggs PJ, Carter PE, Holland BM, French NP. Quantification of historical livestock importation into New Zealand 1860–1979. New Zealand Veterinary Journal. 2014;62(6):309–14. 10.1080/00480169.2014.914861 24869627

[42] Al Mawly J, Grinberg A, Velathanthiri N, French N. Cross sectional study of prevalence, genetic diversity and zoonotic potential of Cryptosporidium parvum cycling in New Zealand dairy farms. Parasites & Vectors. 2015;8:240 10.1186/s13071-015-0855-9 25896433 PMC4423479

[43] Glaberman S, Moore JE, Lowery CJ, Chalmers RM, Sulaiman I, Elwin K, et al Three Drinking-Water–Associated Cryptosporidiosis Outbreaks, Northern Ireland. Emerging Infectious Diseases. 2002;8(6):631–3. 10.3201/eid0806.010368 12023922 PMC2738494

[44] Waldron LS, Ferrari BC, Power ML. Glycoprotein 60 diversity in C. hominis and C. parvum causing human cryptosporidiosis in NSW, Australia. Experimental Parasitology. 2009;122(2):124–7. 10.1016/j.exppara.2009.02.006. 19233175

[45] Kearse M, Moir R, Wilson A, Stones-Havas S, Cheung M, Sturrock S. Geneious Basic: an integrated and extendable desktop software platform for the organization and analysis of sequence data. Bioinformatics. 2012;28(12):1647–9. 10.1093/bioinformatics/bts199 22543367 PMC3371832

[46] Xiao L. Molecular epidemiology of cryptosporidiosis: An update. Experimental Parasitology. 2010;124(1):80–9. 10.1016/j.exppara.2009.03.018. 19358845

[47] Liu K, Warnow TJ, Holder MT, Nelesen SM, Yu J, Stamatakis AP, et al SATé-II: Very fast and accurate simultaneous estimation of multiple sequence alignments and phylogenetic trees. Systematic Biology. 2012;61(1):90–106. 10.1093/sysbio/syr095 22139466

[48] Wickham H. ggplot2: Elegant graphics for data analysis. New York: Springer-Verlag; 2009.

[49] South A. rworldmap: A new R package for mapping global data. The R Journal. 2011;3(1):35–43.

[50] Zhang J, Ding Q, Huang J. spaa: Species association analysis. R package version 0.2. 1; 2013.

[51] Dixon P. VEGAN, a package of R functions for community ecology. Journal of Vegetation Science. 2003;14(6):927–30. 10.1111/j.1654-1103.2003.tb02228.x

[52] Dove ADM, Cribb TH. Species accumulation curves and their applications in parasite ecology. Trends in Parasitology. 2006;22(12):568–74. 10.1016/j.pt.2006.09.008. 17035087

[53] Excoffier L, Laval G, Schneider S. Arlequin (version 3.0): An integrated software package for population genetics data analysis. Evolutionary Bioinformatics Online. 2005;1:47–50. PMC2658868 19325852

[54] Castresana J. Selection of conserved blocks from multiple alignments for their use in phylogenetic analysis. Molecular Biology and Evolution. 2000;17(4):540–52. 10742046 10.1093/oxfordjournals.molbev.a026334

[55] Widmer G. Meta-analysis of a polymorphic surface glycoprotein of the parasitic protozoa *Cryptosporidium parvum* and *Cryptosporidium hominis*. Epidemiology & Infection. 2009;137(12):1800–8. 10.1017/S0950268809990215 19527551 PMC2783587

[56] Choy SH, Mahdy MAK, Al-Mekhlafi HM, Low VL, Surin J. Population expansion and gene flow in *Giardia duodenalis* as revealed by triosephosphate isomerase gene. Parasites & Vectors. 2015;8(1):1–10. 10.1186/s13071-015-1084-y 26373536 PMC4572684

[57] Tanrıverdi S, Grinberg A, Chalmers RM, Hunter PR, Petrovic Z, Akiyoshi DE, et al Inferences about the global population structures of *Cryptosporidium parvum* and *Cryptosporidium hominis*. Applied and Environmental Microbiology. 2008;74(23):7227–34. 10.1128/AEM.01576-08 18836013 PMC2592928

[58] Cacciò SM, de Waele V, Widmer G. Geographical segregation of *Cryptosporidium parvum* multilocus genotypes in Europe. Infection, Genetics and Evolution. 2015;31(0):245–9. 10.1016/j.meegid.2015.02.008. 25687913

[59] Widmer G, Sullivan S. Genomics and population biology of *Cryptosporidium* species. Parasite Immunology. 2012;34(2–3):61–71. 10.1111/j.1365-3024.2011.01301.x 21595702 PMC3168714

[60] Meirmans PG, Hedrick PW. Assessing population structure: FST and related measures. Molecular Ecology Resources. 2011;11(1):5–18. 10.1111/j.1755-0998.2010.02927.x 21429096

[61] Meirmans PG. Using the AMOVA framework to estimate a standardized genetic differentiation measure. Evolution. 2006;60(11):2399–402. 17236430

[62] Fayer R, Xiao L. *Cryptosporidium* and Cryptosporidiosis, Second Edition: CRC Press; 2008.

[63] Siripattanapipong S, Leelayoova S, Mungthin M, Thompson RCA, Boontanom P, Saksirisampant W, et al Clonal diversity of the glutamate dehydrogenase gene in *Giardia duodenalis* from Thai Isolates: evidence of genetic exchange or Mixed Infections? BMC Microbiology. 2011;11:206- 10.1186/1471-2180-11-206 21933419 PMC3191338

[64] Heyworth MF. *Giardia duodenalis* genetic assemblages and hosts. Parasite. 2016;23:13–7. 10.1051/parasite/2016013 26984116 PMC4794627

[65] Koehler AV, Whipp MJ, Haydon SR, Gasser RB. *Cryptosporidium cuniculus*—new records in human and kangaroo in Australia. Parasites & Vectors. 2014;7:492 10.1186/s13071-014-0492-8 25359081 PMC4221722

[66] Chalmers R, Robinson G, Elwin K, Hadfield S, Xiao L, Ryan U, et al *Cryptosporidium* sp. rabbit genotype, a newly identified human pathogen. Emerging Infectious Diseases. 2009;15(5):829–30. 10.3201/eid1505.081419 19402985 PMC2687022

[67] Garcia-R JC, Hayman DTS. Origin of a major infectious disease in vertebrates: The timing of *Cryptosporidium* evolution and its hosts. Parasitology. 2016;143(13):1683–90. 10.1017/S0031182016001323 27573060

[68] Ryan U, Fayer R, Xiao L. *Cryptosporidium* species in humans and animals: current understanding and research needs. Parasitology. 2014;141(13):1667–85. 10.1017/S0031182014001085 25111501

[69] Li N, Xiao L, Alderisio K, Elwin K, Cebelinski E, Chalmers R, et al Subtyping *Cryptosporidium ubiquitum*,a Zoonotic Pathogen Emerging in Humans. Emerging Infectious Diseases. 2014;20(2):217–24. 10.3201/eid2002.121797 24447504 PMC3901490

[70] Shrestha RD, Grinberg A, Dukkipati VSR, Pleydell EJ, Prattley DJ, French NP. Infections with multiple *Cryptosporidium* species and new genetic variants in young dairy calves on a farm located within a drinking water catchment area in New Zealand. Veterinary Parasitology. 2014;202(3–4):287–91. 10.1016/j.vetpar.2014.03.034. 24780161

[71] Dyachenko V, Kuhnert Y, Schmaeschke R, Etzold M, Pantchev N, Daugschies A. Occurrence and molecular characterization of *Cryptosporidium* spp. genotypes in European hedgehogs (Erinaceus europaeus L.) in Germany. Parasitology. 2010;137(02):205–16. 10.1017/S0031182009991089 19765339

[72] Ren X, Zhao J, Zhang L, Ning C, Jian F, Wang R, et al *Cryptosporidium tyzzeri* n. sp. (Apicomplexa: Cryptosporidiidae) in domestic mice (Mus musculus). Experimental Parasitology. 2012;130(3):274–81. 10.1016/j.exppara.2011.07.012. 21803038

[73] Xu F, Jerlstrom-Hultqvist J, Andersson JO. Genome-wide analyses of recombination suggest that Giardia intestinalis assemblages represent different species. Mol Biol Evol. 2012;29 10.1093/molbev/mss107 22474166

[74] Slapeta J. Centenary of the genus *Cryptosporidium*: from morphological to molecular species identification In: Ortega-Pierres MG, Caccio S, Fayer R, Mank T, Smith H, Thompson RCA, editors. *Giardia* and *Crytosporidium*: CABI Publishing; 2009 p. 31–50.

[75] Takumi K, Cacciò SM, Giessen Jvd, Xiao L, Sprong H. Hypothesis: *Cryptosporidium* genetic diversity mirrors national disease notification rate. Parasites & Vectors. 2015;8(308):1–7. 26048280 10.1186/s13071-015-0921-3 PMC4460647

[76] Sprong H, Cacciò SM, van der Giessen JWB, on behalf of the Zn, partners. Identification of Zoonotic Genotypes of *Giardia duodenalis*. PLoS Negl Trop Dis. 2009;3(12):e558 10.1371/journal.pntd.0000558 19956662 PMC2777335

[77] Feng X, Rich SM, Tzipori S, Widmer G. Experimental evidence for genetic recombination in the opportunistic pathogen *Cryptosporidium parvum*. Molecular and Biochemical Parasitology. 2002;119(1):55–62. 10.1016/S0166-6851(01)00393-0. 11755186

[78] Kosuwin R, Putaporntip C, Pattanawong U, Jongwutiwes S. Clonal diversity in *Giardia duodenalis* isolates from Thailand: evidences for intragenic recombination and purifying selection at the beta giardin locus. Gene. 2010;449:1–8. 10.1016/j.gene.2009.09.010 19796671

[79] Feng Y, Torres E, Li N, Wang L, Bowman D, Xiao L. Population genetic characterisation of dominant *Cryptosporidium parvum* subtype IIaA15G2R1. International Journal for Parasitology. 2013;43(14):1141–7. 10.1016/j.ijpara.2013.09.002. 24126186

[80] Hira KG, Mackay MR, Hempstead AD, Ahmed S, Karim MM, O'Connor RM, et al Genetic diversity of *Cryptosporidium* spp. from Bangladeshi children. Journal of Clinical Microbiology. 2011;49(6):2307–10. 10.1128/JCM.00164-11 21471344 PMC3122776

[81] Ajjampur SSR, Liakath FB, Kannan A, Rajendran P, Sarkar R, Moses PD, et al Multisite study of cryptosporidiosis in children with diarrhea in India. Journal of Clinical Microbiology. 2010;48(6):2075–81. 10.1128/JCM.02509-09 20392919 PMC2884513

[82] Ramírez JD, Heredia RD, Hernández C, León CM, Moncada LI, Reyes P, et al Molecular diagnosis and genotype analysis of *Giardia duodenalis* in asymptomatic children from a rural area in central Colombia. Infection, Genetics and Evolution. 2015;32:208–13. 10.1016/j.meegid.2015.03.015. 25795384

[83] Morse T D, Nichols R A B, Grimason A M, Campbell B M, Tembo K C, Smith H V. Incidence of cryptosporidiosis species in paediatric patients in Malawi. Epidemiology and Infection. 2007;135(8):1307–15. 10.1017/S0950268806007758 17224087 PMC2870691

[84] Abe N, Matsubayashi M, Kimata I, Iseki M. Subgenotype analysis of *Cryptosporidium parvum* isolates from humans and animals in Japan using the 60-kDa glycoprotein gene sequences. Parasitol Res. 2006;99(3):303–5. 10.1007/s00436-006-0140-0 16565816

[85] Li W, Kiulia NM, Mwenda JM, Nyachieo A, Taylor MB, Zhang X, et al *Cyclospora papionis*, *Cryptosporidium hominis*, and human-pathogenic *Enterocytozoon bieneusi* in captive baboons in Kenya. Journal of Clinical Microbiology. 2011;49(12):4326–9. 10.1128/JCM.05051-11 21956988 PMC3232936

[86] Lal A, Dobbins T, Bagheri N, Baker MG, French NP, Hales S. Cryptosporidiosis risk in New Zealand children under 5 years old is greatest in areas with high dairy cattle densities. EcoHealth. 2016;13(4):652–60. 10.1007/s10393-016-1187-8 27766441

[87] Zahedi A, Monis P, Aucote S, King B, Paparini A, Jian F, et al Zoonotic *Cryptosporidium s*pecies in animals inhabiting Sydney water catchments. PLOS ONE. 2016;11(12):e0168169 10.1371/journal.pone.0168169 27973572 PMC5156390

