# Supplementary figures and images for: Local and global genetic diversity of protozoan parasites: Spatial distribution of Cryptosporidium and Giardia genotypes
Source: PLoS Negl Trop Dis. 2017 Jul 13;11(7):e0005736. doi: 10.1371/journal.pntd.0005736 (PMC5526614; doi:10.1371/journal.pntd.0005736)

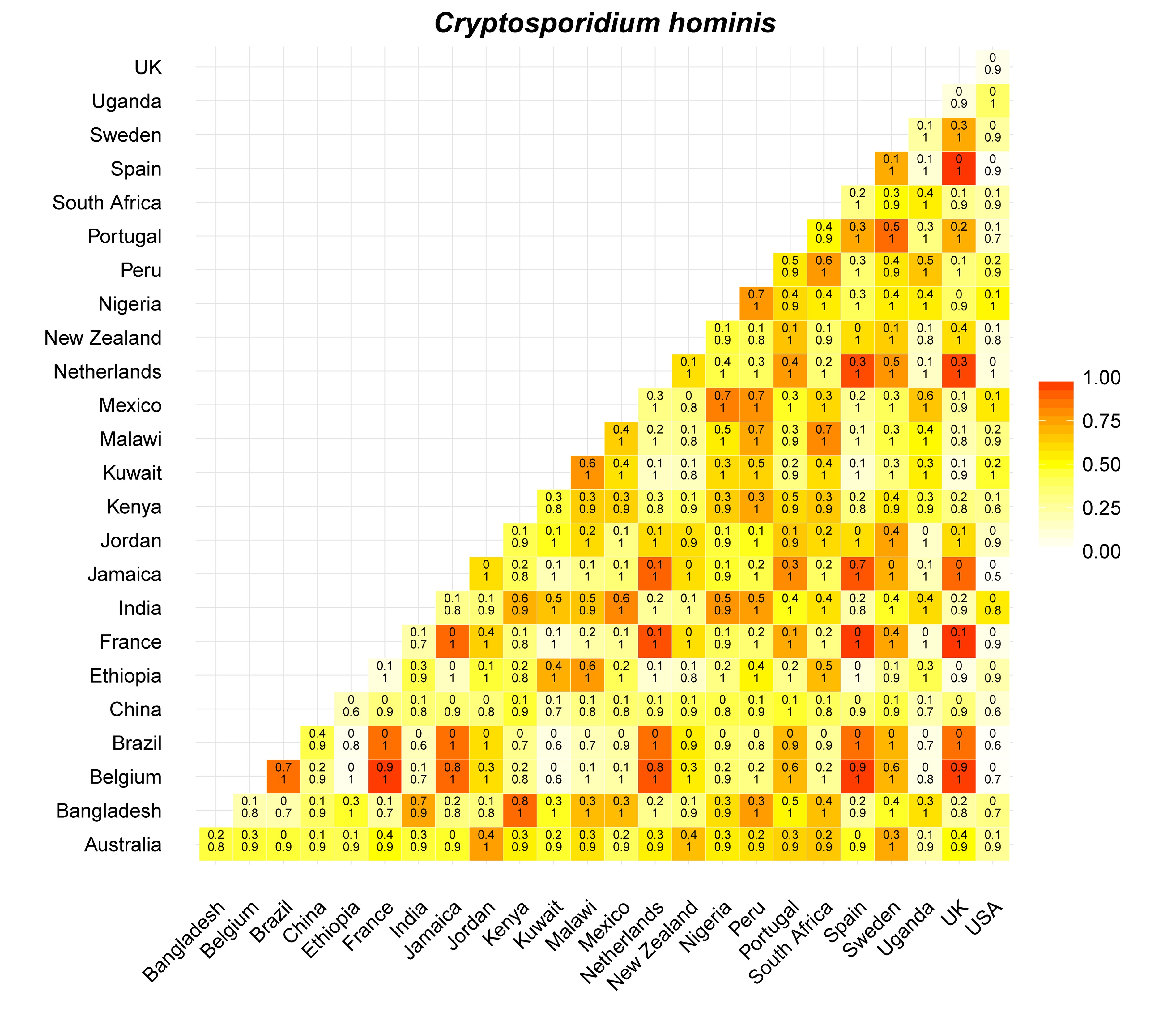

Supplement: S1 Fig — Highly similar countries are showed in darker colours and 95% lower (above) and upper (below) bootstrapped confidence limits in numbers within each box. (TIF) [file pntd.0005736.s001.tif]

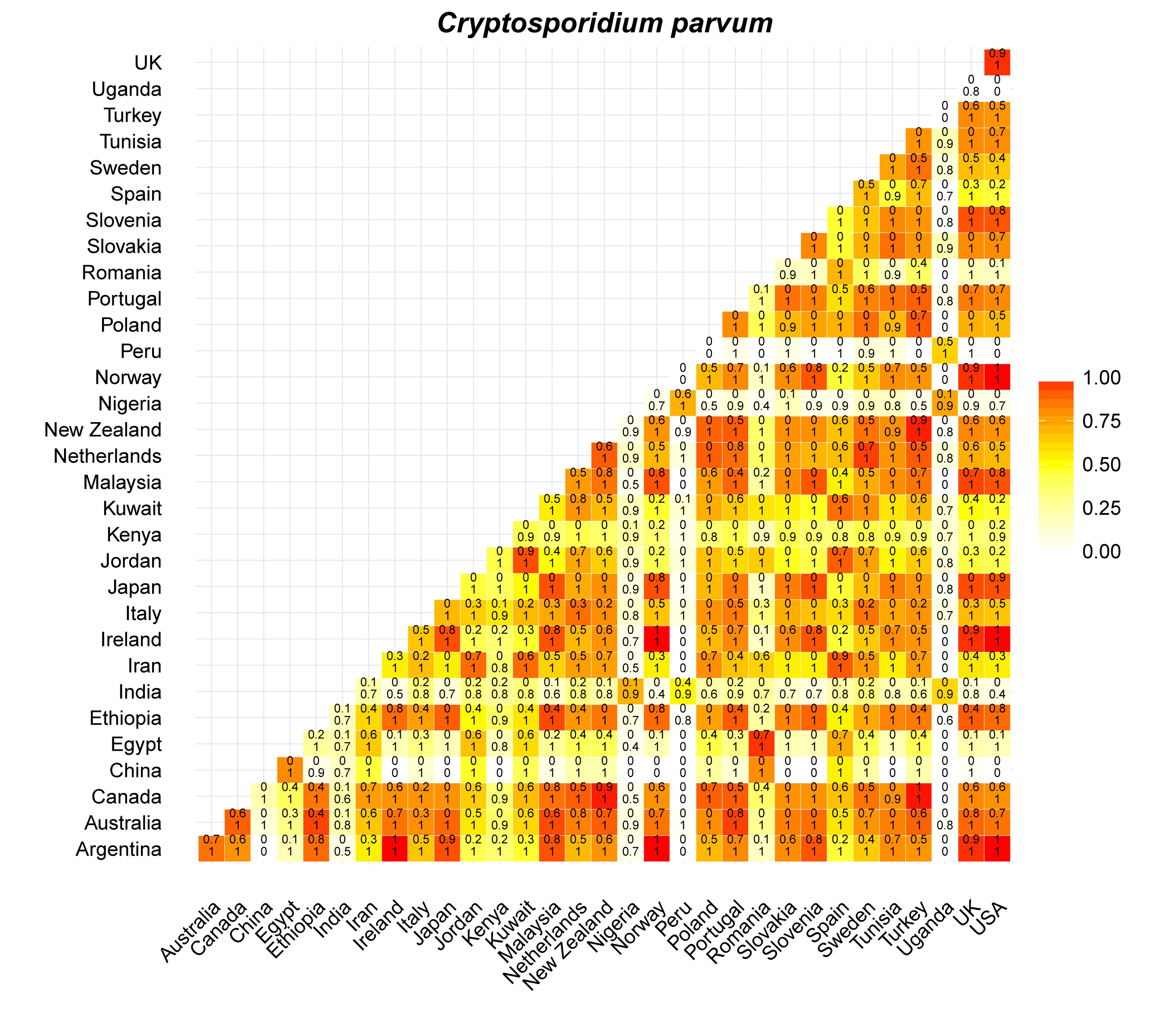

Supplement: S2 Fig — Highly similar countries are showed in darker colours and 95% lower (above) and upper (below) bootstrapped confidence limits in numbers within each box. (TIF) [file pntd.0005736.s002.tif]

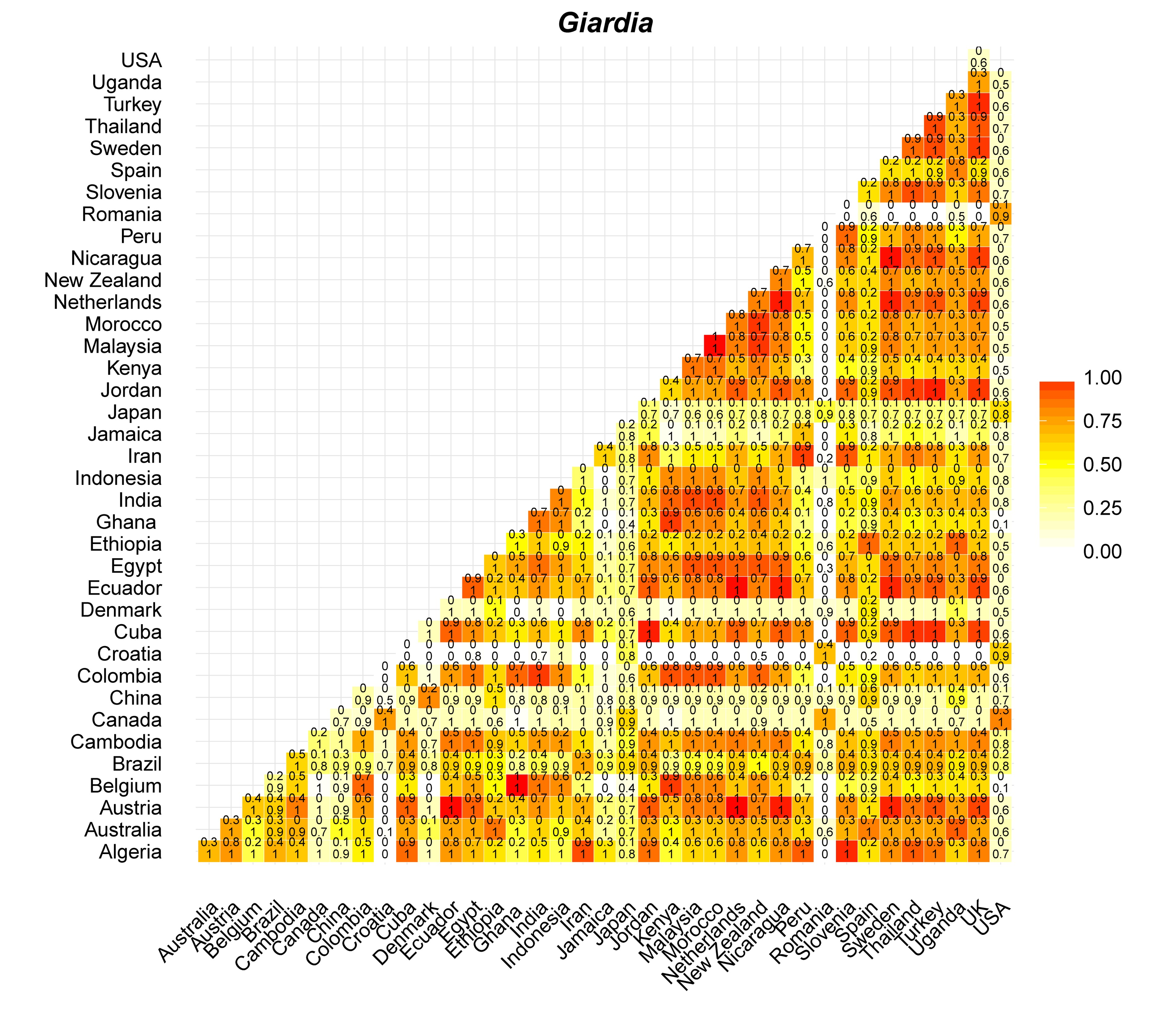

Supplement: S3 Fig — Highly similar countries are showed in darker colours and 95% lower (above) and upper (below) bootstrapped confidence limits in numbers within each box. (TIF) [file pntd.0005736.s003.tif]

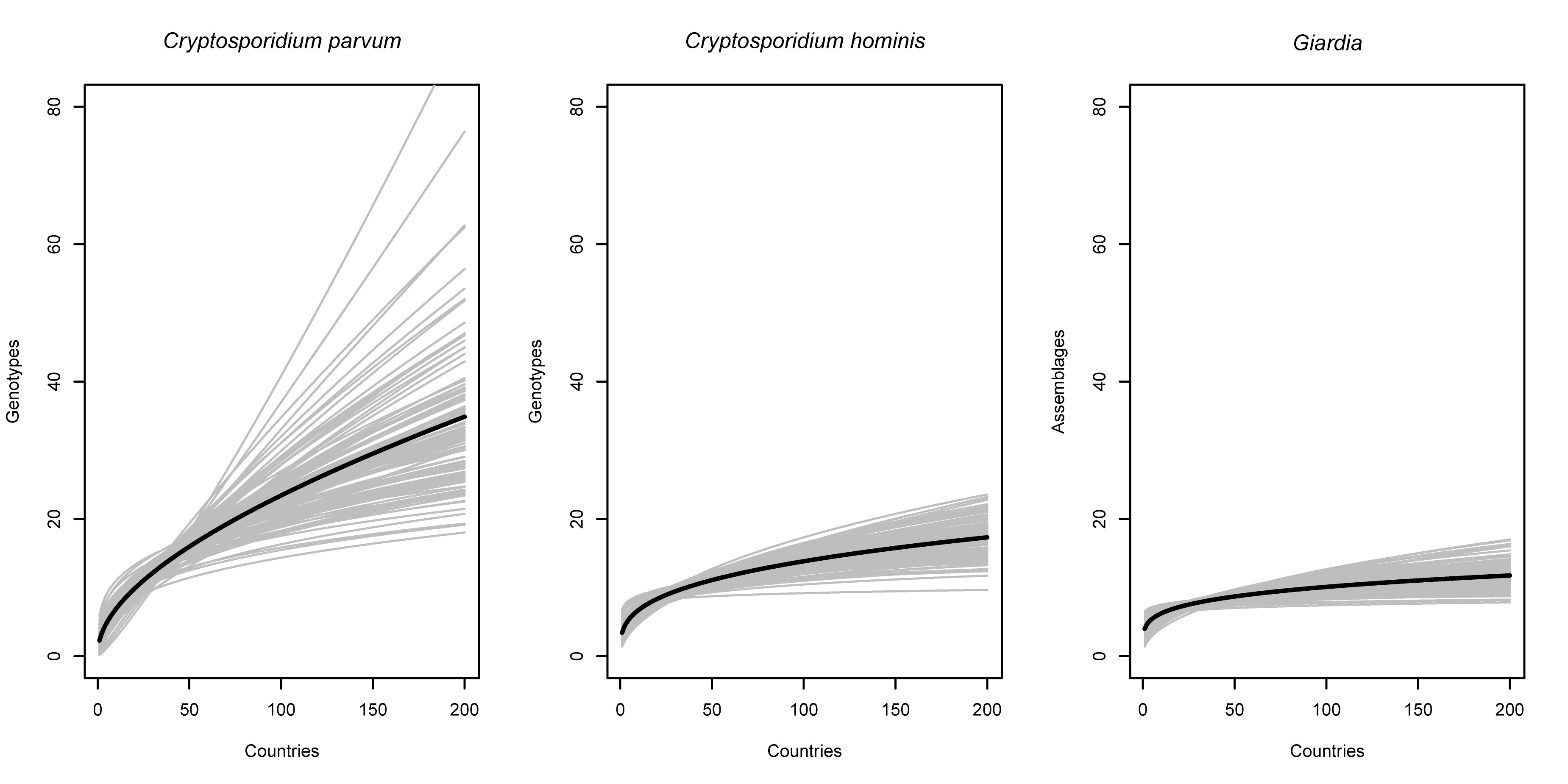

Supplement: S4 Fig — (TIF) [file pntd.0005736.s004.tif]

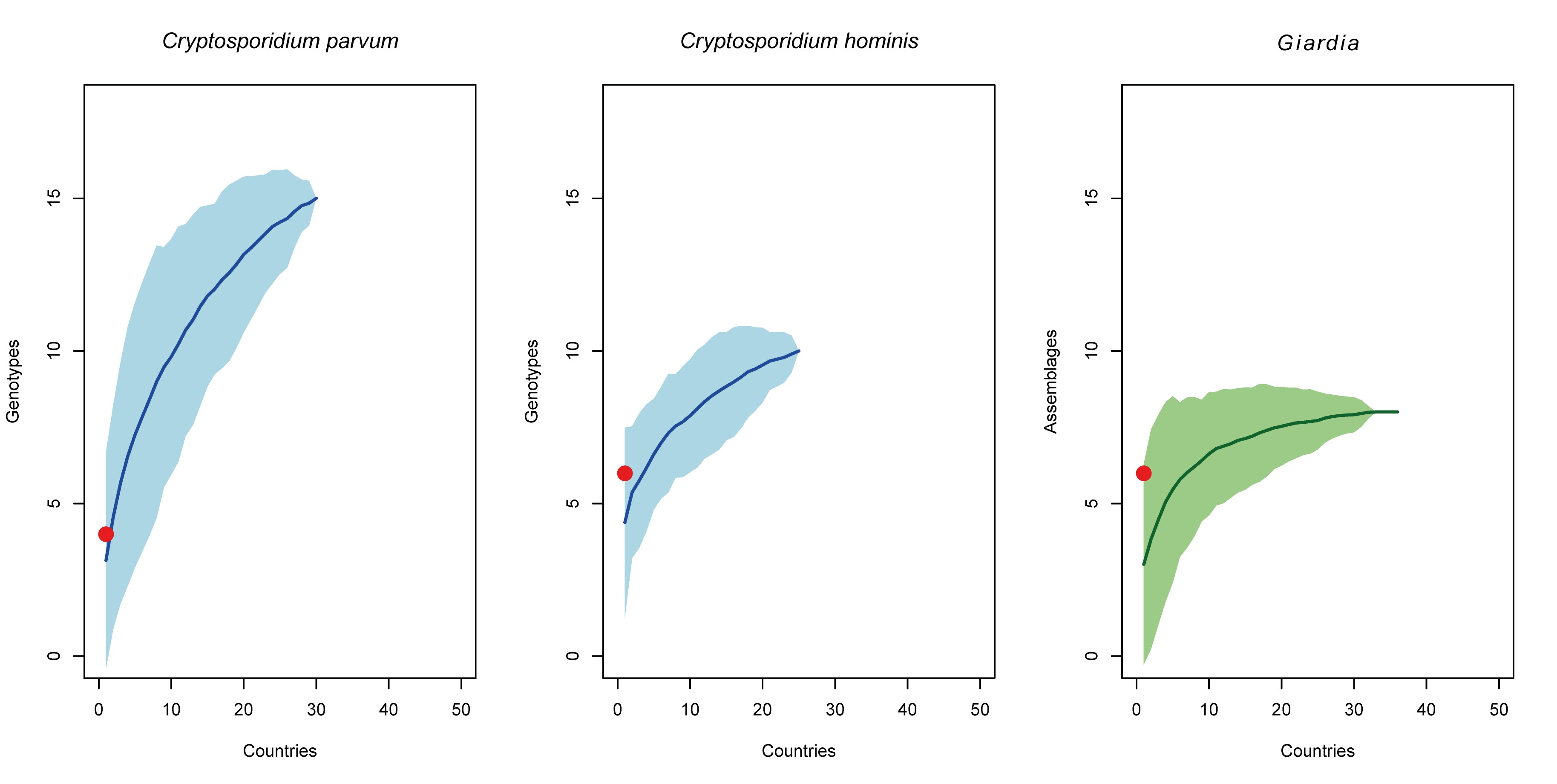

Supplement: S5 Fig — For each curve the lighter shaded region shows 95% confidence intervals and the red square correspond to the number of genotypes for each species found in New Zealand. (TIF) [file pntd.0005736.s005.tif]
