# Supplementary material for: Local and global genetic diversity of protozoan parasites: Spatial distribution of Cryptosporidium and Giardia genotypes
Source: PLoS Negl Trop Dis. 2017 Jul 13;11(7):e0005736. doi: 10.1371/journal.pntd.0005736 (PMC5526614; doi:10.1371/journal.pntd.0005736)
Supplement: S1 Table — (DOCX) [file pntd.0005736.s006.docx]

S1 Table. Primers for PCR and DNA sequencing employed in this study.

| **Locus** | **Position** | **Primer name** | **Sequence** | **Reference** |
| --- | --- | --- | --- | --- |
| gp60 | External | AL3531F | ATAGTCTCCGCTGTATTC | Glaberman et al 2002 |
|  | External | AL3534R | GCAGAGGAACCAGCATC |  |
|  | Internal | AL3532F | TCCGCTGTATTCTCAGCC |  |
|  | Internal | AL3535R | GGAAGGAACGATGTATCT |  |
|  | External | S60.F728 | ACCACATTTTACCCACACATC | Waldron et al 2009 |
|  | External | S60.OutR | TCCTCACTCGATCTAGCTCA |  |
|  | Internal | S60.ATGF | ATGAGATTGTCGCTCATTATCG |  |
|  | Internal | S60.StopR | TTACAACACGAATAAGGCTGC |  |
| *gdh* | External | GDHeF | TCAACGTYAAYCGYGGYTTCCGT | Read et al 2004 |
|  | External/Internal | GDHiR | GTTRTCCTTGCACATCTCC |  |
|  | Internal | GDHiF | CAGTACAACTCYGCTCTCGG |  |
