# Supplementary material for: Local and global genetic diversity of protozoan parasites: Spatial distribution of Cryptosporidium and Giardia genotypes
Source: PLoS Negl Trop Dis. 2017 Jul 13;11(7):e0005736. doi: 10.1371/journal.pntd.0005736 (PMC5526614; doi:10.1371/journal.pntd.0005736)
Supplement: S2 Table — (DOCX) [file pntd.0005736.s007.docx]

S2 Table. Species, sample ID, haplotype number, host, genotypes, number of sequences per haplotype and GenBank accession numbers of data included in this study.

| **Species** | **Sample ID** | **Haplotype** | **Host** | **Genotype** | **N** | **GenBank accession number** |
| --- | --- | --- | --- | --- | --- | --- |
| *Cryptosporidium cuniculus* | 6584 | 1 | Human | Vb | 2 | KY123918 |
|  | 3132 | 2 | Human |  | 1 | KY123919 |
|  | 10171 | 3 | Human |  | 1 | KY123920 |
| *C. erinacei* | 1823 | 1 | Human | XIII | 1 | KY123921 |
|  | 10380 | 2 | Human |  | 1 | KY123922 |
| *C. hominis* | 10041 | 1 | Human | Ia | 2 | KY123923 |
|  | 10296 | 2 | Human |  | 2 | KY123924 |
|  | 5527 | 3 | Human | Id | 6 | KY123925 |
|  | 5191 | 4 | Human |  | 2 | KY123926 |
|  | 6582 | 5 | Human |  | 1 | KY123927 |
|  | 10521 | 6 | Human | Ie | 6 | KY123928 |
|  | 10324 | 7 | Human |  | 4 | KY123929 |
|  | 5740 | 8 | Human | Ig | 67 | KY123930 |
|  | 6769 | 9 | Human |  | 1 | KY123931 |
|  | 4040 | 10 | Human |  | 2 | KY123932 |
|  | 6761 | 11 | Human |  | 1 | KY123933 |
|  | 7018 | 12 | Human |  | 1 | KY123934 |
|  | 9176 | 13 | Human |  | 9 | KY123935 |
|  | 10073 | 14 | Human | Ib | 2 | KY123936 |
|  | 5154 | 15 | Human |  | 1 | KY123937 |
|  | 3527 | 16 | Human |  | 1 | KY123938 |
|  | 4813 | 17 | Human |  | 1 | KY123939 |
|  | 5813 | 18 | Human |  | 126 | KY123940 |
|  | 9400 | 19 | Human |  | 1 | KY123941 |
|  | 9111 | 20 | Human | If | 1 | KY123942 |
|  | 7181 | 21 | Human |  | 1 | KY123943 |
|  | 6528 | 22 | Human |  | 1 | KY123944 |
|  | 6886 | 23 | Human |  | 1 | KY123945 |
|  | 5059 | 24 | Human |  | 1 | KY123946 |
| *C. parvum* | 4642 | 1 | Human | IIa | 138 | KY123947 |
|  | 6694 | 2 | Human |  | 1 | KY123948 |
|  | 2904 | 3 | Human |  | 1 | KY123949 |
|  | 2769 | 4 | Human |  | 1 | KY123950 |
|  | 9917 | 5 | Human |  | 13 | KY123951 |
|  | 9907 | 6 | Human |  | 2 | KY123952 |
|  | 9354 | 7 | Human |  | 1 | KY123953 |
|  | 9902 | 8 | Human |  | 3 | KY123954 |
|  | 4987 | 9 | Human |  | 1 | KY123955 |
|  | 10374 | 10 | Human |  | 1 | KY123956 |
|  | 9576 | 11 | Human |  | 3 | KY123957 |
|  | 5581 | 12 | Human |  | 1 | KY123958 |
|  | 6690 | 13 | Human |  | 3 | KY123959 |
|  | 4588 | 14 | Human |  | 32 | KY123960 |
|  | 4088 | 15 | Human |  | 2 | KY123961 |
|  | 3044 | 16 | Human |  | 1 | KY123962 |
|  | 5386 | 17 | Human |  | 1 | KY123963 |
|  | 5978 | 18 | Human |  | 1 | KY123964 |
|  | 9994 | 19 | Human |  | 10 | KY123965 |
|  | 4732 | 20 | Human |  | 1 | KY123966 |
|  | 9985 | 21 | Human | IId | 29 | KY123967 |
|  | 10178 | 22 | Human |  | 1 | KY123968 |
|  | 10042 | 23 | Human |  | 11 | KY123969 |
|  | 4041 | 24 | Human |  | 1 | KY123970 |
|  | 7377 | 25 | Human |  | 1 | KY123971 |
|  | 8803 | 26 | Human |  | 2 | KY123972 |
|  | 5188 | 27 | Human |  | 1 | KY123973 |
|  | 9999 | 28 | Human |  | 1 | KY123974 |
|  | 4009 | 29 | Human |  | 1 | KY123975 |
|  | 5704 | 30 | Human |  | 2 | KY123976 |
|  | 806 | 31 | Human |  | 1 | KY123977 |
|  | 4408 | 32 | Human |  | 1 | KY123978 |
|  | 6723 | 33 | Human |  | 2 | KY123979 |
|  | 10216 | 34 | Human |  | 1 | KY123980 |
|  | 9127 | 35 | Human |  | 1 | KY123981 |
|  | 10049 | 36 | Human |  | 2 | KY123982 |
|  | 10173 | 37 | Human |  | 1 | KY123983 |
|  | 9115 | 38 | Human |  | 6 | KY123984 |
|  | 3230 | 39 | Human |  | 1 | KY123985 |
|  | 5058 | 40 | Human | IIc | 3 | KY123986 |
|  | 5832 | 41 | Human | IIe | 1 | KY123987 |
|  | 4642_c | 1 | Cattle | IIa | 23 | KY123988 |
|  | 4588_c | 14 | Cattle | IIa | 18 | KY123989 |
|  | 9985_c | 21 | Cattle | IId | 1 | KY123990 |
|  | 9115_c | 38 | Cattle | IId | 1 | KY123991 |
|  | 4642_s | 1 | Sheep | IIa | 1 | KY123992 |
|  | 4588_s | 14 | Sheep | IIa | 1 | KY123993 |
|  |  |  |  |  |  |  |
| *Giardia intestinalis* | 10112_A | 1 | Human | A | 1 | KY123994 |
|  | 9856 | 2 | Human |  | 2 | KY123995 |
|  | 9856_cat | 2 | Cat |  | 1 | KY123996 |
|  | 10129 | 3 | Human |  | 6 | KY123997 |
|  | 10129_cattle | 3 | Cattle |  | 1 | KY123998 |
|  | 10146 | 4 | Human |  | 6 | KY123999 |
|  | 10146_duck | 4 | Duck |  | 2 | KY124000 |
|  | 10210 | 5 | Human |  | 235 | KY124001 |
|  | 10210_cat | 5 | Cat |  | 1 | KY124002 |
|  | 10210_dog | 5 | Dog |  | 1 | KY124003 |
|  | 10210_gibbon | 5 | Gibbon |  | 1 | KY124004 |
|  | 6540 | 6 | Human |  | 1 | KY124005 |
|  | 3184 | 7 | Human |  | 3 | KY124006 |
|  | 9085 | 8 | Human |  | 1 | KY124007 |
|  | 7197 | 9 | Human |  | 1 | KY124008 |
|  | 9544 | 10 | Human |  | 1 | KY124009 |
|  | 10402 | 11 | Human |  | 1 | KY124010 |
|  | 9481_B | 12 | Human | B | 63 | KY124011 |
|  | 5924 | 13 | Human |  | 750 | KY124012 |
|  | 5924_cattle | 13 | Cattle |  | 2 | KY124013 |
|  | 5924_dog | 13 | Dog |  | 2 | KY124014 |
|  | 5924_lemur | 13 | Lemur |  | 1 | KY124015 |
|  | 5924_meerkat | 13 | Meerkat |  | 1 | KY124016 |
|  | 5924_possum | 13 | Possum |  | 1 | KY124017 |
|  | 5924_spider | 13 | Spider Monkey |  | 1 | KY124018 |
|  | 10707_porcupine | 14 | Porcupine |  | 4 | KY124019 |
|  | 10707 | 14 | Human |  | 1 | KY124020 |
|  | 6612_duck | 15 | Duck |  | 2 | KY124021 |
|  | 6609_duck | 16 | Duck |  | 2 | KY124022 |
|  | 5622_bonnet | 17 | Bonnet macaque |  | 1 | KY124023 |
|  | 6948 | 18 | Human |  | 1 | KY124024 |
|  | 6948_duck | 18 | Duck |  | 1 | KY124025 |
|  | 6973 | 19 | Human |  | 1 | KY124026 |
|  | 6973_cattle | 19 | Cattle |  | 1 | KY124027 |
|  | 8963 | 20 | Human |  | 1 | KY124028 |
|  | 8963_cattle | 20 | Cattle |  | 1 | KY124029 |
|  | 8963_duck | 20 | Duck |  | 1 | KY124030 |
|  | 9088 | 21 | Human |  | 1 | KY124031 |
|  | 9088_duck | 21 | Duck |  | 1 | KY124032 |
|  | 9309 | 22 | Human |  | 1 | KY124033 |
|  | 9309_cattle | 22 | Cattle |  | 1 | KY124034 |
|  | 9336 | 23 | Human |  | 1 | KY124035 |
|  | 9336_cattle | 23 | Cattle |  | 1 | KY124036 |
|  | 9336_duck | 23 | Duck |  | 1 | KY124037 |
|  | 9102 | 24 | Human |  | 1 | KY124038 |
|  | 5629 | 25 | Human |  | 8 | KY124039 |
|  | 6488 | 26 | Human |  | 7 | KY124040 |
|  | 5064 | 27 | Human |  | 6 | KY124041 |
|  | 4739 | 28 | Human |  | 5 | KY124042 |
|  | 9659 | 29 | Human |  | 5 | KY124043 |
|  | 9292 | 30 | Human |  | 4 | KY124044 |
|  | 6350 | 31 | Human |  | 4 | KY124045 |
|  | 5633 | 32 | Human |  | 5 | KY124046 |
|  | 9579 | 33 | Human |  | 5 | KY124047 |
|  | 8888 | 34 | Human |  | 5 | KY124048 |
|  | 9073 | 35 | Human |  | 3 | KY124049 |
|  | 9699 | 36 | Human |  | 3 | KY124050 |
|  | 5646 | 37 | Human |  | 11 | KY124051 |
|  | 10107 | 38 | Human |  | 4 | KY124052 |
|  | 8970 | 39 | Human |  | 6 | KY124053 |
|  | 9264 | 40 | Human |  | 6 | KY124054 |
|  | 8818 | 41 | Human |  | 4 | KY124055 |
|  | 8752 | 42 | Human |  | 4 | KY124056 |
|  | 9573 | 43 | Human |  | 4 | KY124057 |
|  | 6681 | 44 | Human |  | 31 | KY124058 |
|  | 9826 | 45 | Human |  | 14 | KY124059 |
|  | 3592 | 46 | Human |  | 7 | KY124060 |
|  | 3923 | 47 | Human |  | 7 | KY124061 |
|  | 3922 | 48 | Human |  | 4 | KY124062 |
|  | 3951 | 49 | Human |  | 3 | KY124063 |
|  | 10441 | 50 | Human |  | 5 | KY124064 |
|  | 9936 | 51 | Human |  | 5 | KY124065 |
|  | 3617 | 52 | Human |  | 5 | KY124066 |
|  | 5053 | 53 | Human |  | 5 | KY124067 |
|  | 9642 | 54 | Human |  | 6 | KY124068 |
|  | 9674 | 55 | Human |  | 6 | KY124069 |
|  | 6173 | 56 | Human |  | 6 | KY124070 |
|  | 10325 | 57 | Human |  | 5 | KY124071 |
|  | 10032 | 58 | Human |  | 5 | KY124072 |
|  | 10011 | 59 | Human |  | 4 | KY124073 |
|  | 9297 | 60 | Human |  | 4 | KY124074 |
|  | 3108 | 61 | Human |  | 4 | KY124075 |
|  | 6442 | 62 | Human |  | 6 | KY124076 |
|  | 9961 | 63 | Human |  | 5 | KY124077 |
|  | 9484 | 64 | Human |  | 5 | KY124078 |
|  | 9831 | 65 | Human |  | 6 | KY124079 |
|  | 7114 | 66 | Human |  | 6 | KY124080 |
|  | 9703 | 67 | Human |  | 6 | KY124081 |
|  | 10057 | 68 | Human |  | 5 | KY124082 |
|  | 7324 | 69 | Human |  | 5 | KY124083 |
|  | 3128 | 70 | Human |  | 5 | KY124084 |
|  | 7284 | 71 | Human |  | 3 | KY124085 |
|  | 9662 | 72 | Human |  | 2 | KY124086 |
|  | 9648 | 73 | Human |  | 2 | KY124087 |
|  | 9660 | 74 | Human |  | 8 | KY124088 |
|  | 6872 | 75 | Human |  | 5 | KY124089 |
|  | 8967 | 76 | Human |  | 5 | KY124090 |
|  | 7334 | 77 | Human |  | 5 | KY124091 |
|  | 5672 | 78 | Human |  | 5 | KY124092 |
|  | 9077 | 79 | Human |  | 6 | KY124093 |
|  | 9144 | 80 | Human |  | 6 | KY124094 |
|  | 10304 | 81 | Human |  | 6 | KY124095 |
|  | 3540 | 82 | Human |  | 6 | KY124096 |
|  | 9533 | 83 | Human |  | 6 | KY124097 |
|  | 9613 | 84 | Human |  | 5 | KY124098 |
|  | 9825 | 85 | Human |  | 5 | KY124099 |
|  | 9549 | 86 | Human |  | 5 | KY124100 |
|  | 9603 | 87 | Human |  | 4 | KY124101 |
|  | 6881_C_dog | 88 | Dog | C | 3 | KY124102 |
|  | 5534_D_dog | 89 | Dog | D | 4 | KY124103 |
|  | 1013_E_cattle | 90 | Cattle | E | 3 | KY124104 |
|  | 1013_E_sheep | 90 | Sheep |  | 9 | KY124105 |
|  | 4706_cattle | 91 | Cattle |  | 2 | KY124106 |
|  | 4706_sheep | 91 | Sheep |  | 6 | KY124107 |
|  | 4009_cattle | 92 | Cattle |  | 2 | KY124108 |
|  | 4009_sheep | 92 | Sheep |  | 5 | KY124109 |
|  | 1008_cattle | 93 | Cattle |  | 2 | KY124110 |
|  | 1008_sheep | 93 | Sheep |  | 4 | KY124111 |
|  | 954_cattle | 94 | Cattle |  | 1 | KY124112 |
|  | 4076_cattle | 95 | Cattle |  | 1 | KY124113 |
|  | 4083_cattle | 96 | Cattle |  | 1 | KY124114 |
|  | 4087_cattle | 97 | Cattle |  | 1 | KY124115 |
|  | 4844_cattle | 98 | Cattle |  | 1 | KY124116 |
|  | 6565_cattle | 99 | Cattle |  | 1 | KY124117 |
|  | 1006_cattle | 100 | Cattle |  | 1 | KY124118 |
|  | 4390_sheep | 101 | Sheep |  | 3 | KY124119 |
|  | 4265_sheep | 102 | Sheep |  | 2 | KY124120 |
|  | 3665_F_cat | 103 | Cat | F | 2 | KY124121 |
