# Supplementary material for: Local and global genetic diversity of protozoan parasites: Spatial distribution of Cryptosporidium and Giardia genotypes
Source: PLoS Negl Trop Dis. 2017 Jul 13;11(7):e0005736. doi: 10.1371/journal.pntd.0005736 (PMC5526614; doi:10.1371/journal.pntd.0005736)
Supplement: S3 Table — (DOCX) [file pntd.0005736.s008.docx]

S3 Table. Taxa, genotypes, number of sequences, percentage of occurrence and range of hosts of *Cryptosporidium* and *Giardia* species found in this study.

| Species | Genotypes | n | % (95% CI) | Hosts |
| --- | --- | --- | --- | --- |
| *Cryptosporidium hominis* | Ia | 4 | 1.6 (0.6─4.2%) | Human |
|  | Ib | 132 | 54.8 (48.5─60.9%) |  |
|  | Id | 9 | 3.7 (2.0─6.9%) |  |
|  | Ie | 10 | 4.2 (2.3─7.5%) |  |
|  | If | 5 | 2.1 (0.9─4.8%) |  |
|  | Ig | 81 | 33.6 (27.9─39.8%) |  |
| *C. parvum* | IIa | 2 | 78.3 (7.3─82.4%) | Sheep |
|  |  | 41 |  | Cattle |
|  |  | 217 |  | Human |
|  | IIc | 3 | 0.9 (0.3─2.6%) | Human |
|  | IId | 2 | 20.5 (16.5─25.1%) | Cattle |
|  |  | 66 |  | Human |
|  | IIe | 1 | 0.3 (0.1─1.7%) | Human |
| *C. cuniculus* | Vb | 4 | 100 (51─100%) | Human |
| *C. erinacei* | XIIIa | 2 | 100 (34.2─100%) | Human |
| *Giardia intestinalis* | A | 1 | 17.4 (15.6─19.4%) | Cattle |
|  |  | 1 |  | Dog |
|  |  | 2 |  | Duck |
|  |  | 2 |  | Cat |
|  |  | 1 |  | Gibbon |
|  |  | 258 |  | Human |
|  | B | 1 | 79 (76.9─81%) | Spider monkey |
|  |  | 1 |  | Macaque |
|  |  | 2 |  | Dog |
|  |  | 8 |  | Duck |
|  |  | 1 |  | Meerkat |
|  |  | 4 |  | Porcupine |
|  |  | 1 |  | Possum |
|  |  | 1 |  | Lemur |
|  |  | 6 |  | Cattle |
|  |  | 1179 |  | Human |
|  | C | 3 | 0.2 (0.1─0.6%) | Dog |
|  | D | 4 | 0.3 (0.1─0.7%) | Dog |
|  | E | 16 | 3 (2.2─3.9%) | Cattle |
|  |  | 29 |  | Sheep |
|  | F | 2 | 0.1 (0.0─0.5%) | Cat |
