# Supplementary material for: Local and global genetic diversity of protozoan parasites: Spatial distribution of Cryptosporidium and Giardia genotypes
Source: PLoS Negl Trop Dis. 2017 Jul 13;11(7):e0005736. doi: 10.1371/journal.pntd.0005736 (PMC5526614; doi:10.1371/journal.pntd.0005736)
Supplement: S1 References — (PDF) [file pntd.0005736.s011.pdf]

[1-215]


1. Laatamna AE, Wagnerová P, Sak B, Květoňová D, Xiao L, Rost M, et al. Microsporidia and Cryptosporidium in horses and donkeys in Algeria: detection of a novel Cryptosporidium hominis subtype family (Ik) in a horse. Vet Parasitol. 2015;208:135-42.
2. Peralta RHS, Velásquez JN, Cunha FdS, Pantano ML, Sodré FC, Silva Sd, et al. Genetic diversity of Cryptosporidium identified in clinical samples from cities in Brazil and Argentina. Mem Inst Oswaldo Cruz. 2016;111:30-6.
3. Jex AR, Whipp M, Campbell BE, Cacciò SM, Stevens M, Hogg G, et al. A practical and cost-effective mutation scanning-based approach for investigating genetic variation in Cryptosporidium. Electrophoresis. 2007;28:3875-83.
4. Jex AR, Pangasa A, Campbell BE, Whipp M, Hogg G, Sinclair MI, et al. Classification of Cryptosporidium species from patients with sporadic cryptosporidiosis by use of sequence-based multilocus analysis following mutation scanning. J Clin Microbiol. 2008;46:2252-62.
5. Koehler AV, Haydon SR, Jex AR, Gasser RB. Cryptosporidium and Giardia taxa in faecal samples from animals in catchments supplying the city of Melbourne with drinking water (2011 to 2015). Parasit Vectors. 2016;9:315.
6. O'Brien E, McInnes L, Ryan U. Cryptosporidium GP60 genotypes from humans and domesticated animals in Australia, North America and Europe. Exp Parasitol. 2008;118:118-21.
7. Chalmers RM, Ferguson C, Cacciò S, Gasser RB, Abs EL-Osta YG, Heijnen L, et al. Direct comparison of selected methods for genetic categorisation of Cryptosporidium parvum and Cryptosporidium hominis species. Int J Parasitol. 2005;35:397-410.
8. Ng J, Eastwood K, Durrheim D, Massey P, Walker B, Armson A, et al. Evidence supporting zoonotic transmission of Cryptosporidium in rural New South Wales. Exp Parasitol. 2008;119:192-5.
9. Koehler AV, Whipp M, Hogg G, Haydon SR, Stevens MA, Jex AR, et al. First genetic analysis of Cryptosporidium from humans from Tasmania, and identification of a new genotype from a traveller to Bali. Electrophoresis. 2014;35:2600-7.
10. Ebner J, Koehler AV, Robertson G, Bradbury RS, Jex AR, Haydon SR, et al. Genetic analysis of Giardia and Cryptosporidium from people in Northern Australia using PCR-based tools. Infect Genet Evol. 2015;36:389-95.
11. Waldron LS, Ferrari BC, Power ML. Glycoprotein 60 diversity in C. hominis and C. parvum causing human cryptosporidiosis in NSW, Australia. Experimental Parasitology. 2009;122(2):124-7. doi: http://dx.doi.org/10.1016/j.exppara.2009.02.006.
12. Ng J, MacKenzie B, Ryan U. Longitudinal multi-locus molecular characterisation of sporadic Australian human clinical cases of cryptosporidiosis from 2005 to 2008. Exp Parasitol. 2010;125:348-56.
13. Nolan MJ, Jex AR, Koehler AV, Haydon SR, Stevens MA, Gasser RB. Molecular-based investigation of Cryptosporidium and Giardia from animals in water catchments in southeastern Australia. Water Res. 2013;47:1726-40.
14. Hira KG, Mackay MR, Hempstead AD, Ahmed S, Karim MM, O'Connor RM, et al. Genetic diversity of *Cryptosporidium* spp. from Bangladeshi children. Journal of Clinical Microbiology. 2011;49(6):2307-10. doi: 10.1128/jcm.00164-11.
15. Geurden T, Levecke B, Cacció SM, Visser A, De Groote G, Casaert S, et al. Multilocus genotyping of Cryptosporidium and Giardia in non-outbreak related cases of diarrhoea in human patients in Belgium. Parasitology. 2009;136:1161-8.
16. Inácio SV, Widmer G, de Brito RLL, Zucatto AS, de Aquino MCC, Oliveira BCM, et al. First description of Cryptosporidium hominis GP60 genotype IkA20G1 and Cryptosporidium parvum GP60 genotypes IIaA18G3R1 and IIaA15G2R1 in foals in Brazil. Vet Parasitol. 2017;233:48-51.

17. Trotz-Williams LA, Martin DS, Gatei W, Cama V, Peregrine AS, Martin SW, et al. Genotype and subtype analyses of Cryptosporidium isolates from dairy calves and humans in Ontario. Parasitol Res. 2006;99:346-52.
18. Jian F, Liu A, Wang R, Zhang S, Qi M, Zhao W, et al. Common occurrence of Cryptosporidium hominis in horses and donkeys. Infect Genet Evol. 2016;43:261-6.
19. Feng Y, Li N, Duan L, Xiao L. Cryptosporidium genotype and subtype distribution in raw wastewater in Shanghai, China: evidence for possible unique Cryptosporidium hominis transmission. J Clin Microbiol. 2009;47:153-7.
20. Liu X, Xie N, Li W, Zhou Z, Zhong Z, Shen L, et al. Emergence of Cryptosporidium hominis Monkey Genotype II and Novel Subtype Family Ik in the Squirrel Monkey (Saimiri sciureus) in China. PLoS One. 2015;10:e0141450.
21. Karim MR, Zhang S, Jian F, Li J, Zhou C, Zhang L, et al. Multilocus typing of Cryptosporidium spp. and Giardia duodenalis from non-human primates in China. Int J Parasitol. 2014;44:1039-47.
22. Jex AR, Gasser RB. Analysis of the genetic diversity within Cryptosporidium hominis and Cryptosporidium parvum from imported and autochtonous cases of human cryptosporidiosis by mutation scanning. ELECTROPHORESIS. 2008;29(20):4119-29. doi: 10.1002/elps.200800422.
23. Adamu H, Petros B, Zhang G, Kassa H, Amer S, Ye J, et al. Distribution and clinical manifestations of Cryptosporidium species and subtypes in HIV/AIDS patients in Ethiopia. PLoS Negl Trop Dis. 2014;8:e2831.
24. Flecha MJ, Benavides CM, Tissiano G, Tesfamariam A, Cuadros J, de Lucio A, et al. Detection and molecular characterisation of Giardia duodenalis, Cryptosporidium spp. and Entamoeba spp. among patients with gastrointestinal symptoms in Gambo Hospital, Oromia Region, southern Ethiopia. Trop Med Int Health. 2015;20:1213-22.
25. Cohen S, Dalle F, Gallay A, Di Palma M, Bonnin A, Ward HD. Identification of Cpgp40/15 Type Ib as the Predominant Allele in Isolates of Cryptosporidium spp. from a Waterborne Outbreak of Gastroenteritis in South Burgundy, France. Journal of Clinical Microbiology. 2006;44(2):589-91. doi: 10.1128/jcm.44.2.589-591.2006.
26. Gatei W, Das P, Dutta P, Sen A, Cama V, Lal AA. Multilocus sequence typing and genetic structure of cryptosporidium hominis from children in Kolkata, India. Infect Genet Evol. 2007;7. doi: 10.1016/j.meegid.2006.08.006.
27. Ajjampur SSR, Gladstone BP, Selvapandian D, Muliyil JP, Ward H, Kang G. Molecular and spatial epidemiology of cryptosporidiosis in children in a semiurban community in South India. J Clin Microbiol. 2007;45:915-20.
28. Ajjampur SSR, Liakath FB, Kannan A, Rajendran P, Sarkar R, Moses PD, et al. Multisite study of cryptosporidiosis in children with diarrhea in India. Journal of Clinical Microbiology. 2010;48(6):2075-81. doi: 10.1128/jcm.02509-09.
29. Muthusamy D, Rao SS, Ramani S, Monica B, Banerjee I, Abraham OC, et al. Multilocus genotyping of Cryptosporidium sp. isolates from human immunodeficiency virus-infected individuals in South India. J Clin Microbiol. 2006;44:632-4.
30. Sharma P, Sharma A, Sehgal R, Malla N, Khurana S. Genetic diversity of Cryptosporidium isolates from patients in North India. Int J Infect Dis. 2013;17:e601-5.
31. Yadav P, Mirdha BR, Makharia GK, Chaudhry R. Multilocus sequence typing of Cryptosporidium hominis from northern India. Indian J Med Res. 2017;145:102-11.
32. Taghipour N, Nazemalhosseini-Mojarad E, Haghighi A, Rostami-Nejad M, Romani S, Keshavarz A, et al. Molecular epidemiology of cryptosporidiosis in Iranian children, tehran, iran. Iran J Parasitol. 2011;6:41-5.
33. Nazemalhosseini-Mojarad E, Haghighi A, Taghipour N, Keshavarz A, Mohebi SR, Zali MR, et al. Subtype analysis of Cryptosporidium parvum and Cryptosporidium hominis isolates from humans and cattle in Iran. Vet Parasitol. 2011;179:250-2.
34. Del Chierico F, Onori M, Di Bella S, Bordi E, Petrosillo N, Menichella D, et al. Cases of cryptosporidiosis co-infections in AIDS patients: a correlation between clinical presentation and

GP60 subgenotype lineages from aged formalin-fixed stool samples. Ann Trop Med Parasitol. 2011;105:339-49.
35. Wu Z, Nagano I, Boonmars T, Nakada T, Takahashi Y. Intraspecies polymorphism of Cryptosporidium parvum revealed by PCR-restriction fragment length polymorphism (RFLP) and RFLP-single-strand conformational polymorphism analyses. Appl Environ Microbiol. 2003;69:4720-6.
36. Gatei W, Barrett D, Lindo JF, Eldemire-Shearer D, Cama V, Xiao L. Unique Cryptosporidium population in HIV-infected persons, Jamaica. Emerg Infect Dis. 2008;14:841-3.
37. Abe N, Matsubayashi M, Kimata I, Iseki M. Subgenotype analysis of *Cryptosporidium parvum* isolates from humans and animals in Japan using the 60-kDa glycoprotein gene sequences. Parasitol Res. 2006;99(3):303-5. doi: 10.1007/s00436-006-0140-0.
38. Hijjawi N, Mukbel R, Yang R, Ryan U. Genetic characterization of Cryptosporidium in animal and human isolates from Jordan. Vet Parasitol. 2016;228:116-20.
39. Hijjawi N, Ng J, Yang R, Atoum MFM, Ryan U. Identification of rare and novel Cryptosporidium GP60 subtypes in human isolates from Jordan. Exp Parasitol. 2010;125:161-4.
40. Li W, Kiulia NM, Mwenda JM, Nyachieo A, Taylor MB, Zhang X, et al. *Cyclospora papionis, Cryptosporidium hominis*, and human-pathogenic *Enterocytozoon bieneusi* in captive baboons in Kenya. Journal of Clinical Microbiology. 2011;49(12):4326-9. doi: 10.1128/JCM.05051-11. PubMed PMID: PMC3232936.
41. Wanyiri JW, Kanyi H, Maina S, Wang DE, Steen A, Ngugi P, et al. Cryptosporidiosis in HIV/AIDS patients in Kenya: clinical features, epidemiology, molecular characterization and antibody responses. Am J Trop Med Hyg. 2014;91:319-28.
42. Iqbal A, Lim YAL, Surin J, Sim BLH. High Diversity of *Cryptosporidium* Subgenotypes Identified in Malaysian HIV/AIDS Individuals Targeting gp60 Gene. PLoS ONE. 2012;7(2):e31139. doi: 10.1371/journal.pone.0031139.
43. Iqbal J, Khalid N, Hira PR. Cryptosporidiosis in Kuwaiti children: association of clinical characteristics with Cryptosporidium species and subtypes. J Med Microbiol. 2011;60:647-52.
44. Sulaiman IM, Hira PR, Zhou L, Al-Ali FM, Al-Shelahi FA, Shweiki HM. Unique endemicity of cryptosporidiosis in children in Kuwait. J Clin Microbiol. 2005;43. doi: 10.1128/jcm.43.6.2805-2809.2005.
45. Osman M, El Safadi D, Benamrouz S, Guyot K, Dei-Cas E, Aliouat EM, et al. Initial data on the molecular epidemiology of cryptosporidiosis in Lebanon. PLoS One. 2015;10:e0125129.
46. Ghaffari S, Kalantari N. A multi-locus study of cryptosporidium parasites isolated from patients living in Iran, Malawi, Nigeria, the United kingdom, and Vietnam. Iran J Parasitol. 2014;9:79-89.
47. Peng MM, Meshnick SR, Cunliffe NA, Thindwa BDM, Hart CA, Broadhead RL, et al. Molecular epidemiology of cryptosporidiosis in children in Malawi. J Eukaryot Microbiol. 2003;50 Suppl:557-9.
48. Valenzuela O, González-Díaz M, Garibay-Escobar A, Burgara-Estrella A, Cano M, Durazo M, et al. Molecular characterization of *Cryptosporidium* spp. in children from Mexico. PLoS ONE. 2014;9(4):e96128. doi: 10.1371/journal.pone.0096128.
49. Roelfsema JH, Sprong H, Cacciò SM, Takumi K, Kroes M, van Pelt W, et al. Molecular characterization of human Cryptosporidium spp. isolates after an unusual increase in late summer 2012. Parasit Vectors. 2016;9:138.
50. Wielinga PR, de Vries A, van der Goot TH, Mank T, Mars MH, Kortbeek LM, et al. Molecular epidemiology of Cryptosporidium in humans and cattle in The Netherlands. Int J Parasitol. 2008;38:809-17.
51. Abeywardena H, Jex AR, Nolan MJ, Haydon SR, Stevens MA, McAnulty RW, et al. Genetic characterisation of Cryptosporidium and Giardia from dairy calves: discovery of species/genotypes consistent with those found in humans. Infect Genet Evol. 2012;12:1984-93.
52. Ukwah BN, Ezeonu IM, Ezeonu CT, Roellig D, Xiao L. Cryptosporidium species and subtypes in diarrheal children and HIV-infected persons in Ebonyi and Nsukka, Nigeria. J Infect Dev Ctries. 2017;11:173-9.

53. Molloy SF, Smith HV, Kirwan P, Nichols RAB, Asaolu SO, Connelly L, et al. Identification of a high diversity of Cryptosporidium species genotypes and subtypes in a pediatric population in Nigeria. Am J Trop Med Hyg. 2010;82:608-13.
54. Maikai BV, Umoh JU, Lawal IA, Kudi AC, Ejembi CL, Xiao L. Molecular characterizations of Cryptosporidium, Giardia, and Enterocytozoon in humans in Kaduna State, Nigeria. Exp Parasitol. 2012;131:452-6.
55. Koinari M, Karl S, Ng-Hublin J, Lymbery AJ, Ryan UM. Identification of novel and zoonotic Cryptosporidium species in fish from Papua New Guinea. Vet Parasitol. 2013;198:1-9.
56. Koinari M, Lymbery AJ, Ryan UM. Cryptosporidium species in sheep and goats from Papua New Guinea. Exp Parasitol. 2014;141:134-7.
57. Cama VA, Bern C, Roberts J, Cabrera L, Sterling CR, Ortega Y, et al. Cryptosporidium species and subtypes and clinical manifestations in children, Peru. Emerg Infect Dis. 2008;14:1567-74.
58. Alves M, Xiao L, Antunes F, Matos O. Distribution of Cryptosporidium subtypes in humans and domestic and wild ruminants in Portugal. Parasitol Res. 2006;99:287-92.
59. Danišová O, Valenčáková A, Stanko M, Luptáková L, Hatalová E, Čanády A. Rodents as a reservoir of infection caused by multiple zoonotic species/genotypes of C. parvum, C. hominis, C. suis, C. scrofarum, and the first evidence of C. muskrat genotypes I and II of rodents in Europe. Acta Trop. 2017;172:29-35.
60. Soba B, Logar J. Genetic classification of Cryptosporidium isolates from humans and calves in Slovenia. Parasitology. 2008;135:1263-70.
61. Leav BA, Mackay MR, Anyanwu A, O' Connor RM, Cevallos AM, Kindra G, et al. Analysis of Sequence Diversity at the Highly Polymorphic Cpgp40/15 Locus among Cryptosporidium Isolates from Human Immunodeficiency Virus-Infected Children in South Africa. Infection and Immunity. 2002;70(7):3881-90. doi: 10.1128/iai.70.7.3881-3890.2002.
62. de Lucio A, Merino FJ, Martínez-Ruiz R, Bailo B, Aguilera M, Fuentes I, et al. Molecular genotyping and sub-genotyping of Cryptosporidium spp. isolates from symptomatic individuals attending two major public hospitals in Madrid, Spain. Infect Genet Evol. 2016;37:49-56.
63. Insulander M, SilverlÅS C, Lebbad M, Karlsson L, Mattsson JG, Svenungsson B. Molecular epidemiology and clinical manifestations of human cryptosporidiosis in Sweden. Epidemiology and Infection. 2013;141(5):1009-20. Epub 08/09. doi: 10.1017/S0950268812001665.
64. Ben Ayed L, Yang W, Widmer G, Cama V, Ortega Y, Xiao L. Survey and genetic characterization of wastewater in Tunisia for Cryptosporidium spp., Giardia duodenalis, Enterocytozoon bieneusi, Cyclospora cayetanensis and Eimeria spp. J Water Health. 2012;10:431-44.
65. Akiyoshi DE, Tumwine JK, Bakeera-Kitaka S, Tzipori S. Subtype analysis of Cryptosporidium isolates from children in Uganda. J Parasitol. 2006;92:1097-100.
66. Connelly L, Craig BH, Jones B, Alexander CL. Genetic diversity of Cryptosporidium spp. within a remote population of Soay Sheep on St. Kilda Islands, Scotland. Appl Environ Microbiol. 2013;79:2240-6.
67. Chalmers RM, Hadfield SJ, Jackson CJ, Elwin K, Xiao L, Hunter P. Geographic linkage and variation in Cryptosporidium hominis. Emerg Infect Dis. 2008;14:496-8.
68. Feng Y, Tiao N, Li N, Hlavsa M, Xiao L. Multilocus sequence typing of an emerging Cryptosporidium hominis subtype in the United States. J Clin Microbiol. 2014;52:524-30.
69. Feng Y, Lal AA, Li N, Xiao L. Subtypes of Cryptosporidium spp. in mice and other small mammals. Exp Parasitol. 2011;127:238-42.
70. Alyousefi NA, Mahdy MAK, Lim YAL, Xiao L, Mahmud R. First molecular characterization of Cryptosporidium in Yemen. Parasitology. 2013;140:729-34.
71. Benhouda D, Hakem A, Sannella AR, Benhouda A, Cacciò SM. First molecular investigation of Cryptosporidium spp. in young calves in Algeria. Parasite. 2017;24:15.
72. Del Coco VF, Córdoba MA, Bilbao G, de Almeida Castro AP, Basualdo JA, Fayer R, et al. Cryptosporidium parvum GP60 subtypes in dairy cattle from Buenos Aires, Argentina. Res Vet Sci. 2014;96:311-4.

73. Tomazic ML, Maidana J, Dominguez M, Uriarte EL, Galarza R, Garro C, et al. Molecular characterization of Cryptosporidium isolates from calves in Argentina. Vet Parasitol. 2013;198:382-6.
74. Sweeny JPA, Robertson ID, Ryan UM, Jacobson C, Woodgate RG. Impacts of naturally acquired protozoa and strongylid nematode infections on growth and faecal attributes in lambs. Vet Parasitol. 2012;184:298-308.
75. Ng J, Yang R, McCarthy S, Gordon C, Hijjawi N, Ryan U. Molecular characterization of Cryptosporidium and Giardia in pre-weaned calves in Western Australia and New South Wales. Vet Parasitol. 2011;176:145-50.
76. Allison GM, Rogers KA, Borad A, Ahmed S, Karim MM, Kane AV, et al. Antibody responses to the immunodominant Cryptosporidium gp15 antigen and gp15 polymorphisms in a case-control study of cryptosporidiosis in children in Bangladesh. Am J Trop Med Hyg. 2011;85:97-104.
77. Thivierge K, Iqbal A, Dixon B, Dion R, Levesque B, Cantin P, et al. Cryptosporidium hominis Is a Newly Recognized Pathogen in the Arctic Region of Nunavik, Canada: Molecular Characterization of an Outbreak. PLoS Negl Trop Dis. 2016;10:e0004534.
78. Feng Y, Torres E, Li N, Wang L, Bowman D, Xiao L. Population genetic characterisation of dominant *Cryptosporidium parvum* subtype IIaA15G2R1. International Journal for Parasitology. 2013;43(14):1141-7. doi: http://dx.doi.org/10.1016/j.ijpara.2013.09.002.
79. Wang R, Wang H, Sun Y, Zhang L, Jian F, Qi M, et al. Characteristics of Cryptosporidium transmission in preweaned dairy cattle in Henan, China. J Clin Microbiol. 2011;49:1077-82.
80. Du S-Z, Zhao G-H, Shao J-F, Fang Y-Q, Tian G-R, Zhang L-X, et al. Cryptosporidium spp., Giardia intestinalis, and Enterocytozoon bieneusi in Captive Non-Human Primates in Qinling Mountains. Korean J Parasitol. 2015;53:395-402.
81. Lv C, Zhang L, Wang R, Jian F, Zhang S, Ning C, et al. Cryptosporidium spp. in wild, laboratory, and pet rodents in china: prevalence and molecular characterization. Appl Environ Microbiol. 2009;75:7692-9.
82. Zhang W, Wang R, Yang F, Zhang L, Cao J, Zhang X, et al. Distribution and genetic characterizations of Cryptosporidium spp. in pre-weaned dairy calves in Northeastern China's Heilongjiang Province. PLoS One. 2013;8:e54857.
83. Zhao G-H, Du S-Z, Wang H-B, Hu X-F, Deng M-J, Yu S-K, et al. First report of zoonotic Cryptosporidium spp., Giardia intestinalis and Enterocytozoon bieneusi in golden takins (Budorcas taxicolor bedfordi). Infect Genet Evol. 2015;34:394-401.
84. Zhao Z, Dong H, Wang R, Zhao W, Chen G, Li S, et al. Genotyping and subtyping *Cryptosporidium parvum* and *Giardia duodenalis* carried by flies on dairy farms in Henan, China. Parasites & Vectors. 2014;7:190-. doi: 10.1186/1756-3305-7-190. PubMed PMID: PMC4005625.
85. Zhao Z, Wang R, Zhao W, Qi M, Zhao J, Zhang L, et al. Genotyping and subtyping of Giardia and Cryptosporidium isolates from commensal rodents in China. Parasitology. 2015;142:800-6.
86. Liu X, Zhou X, Zhong Z, Zuo Z, Shi J, Wang Y, et al. Occurrence of novel and rare subtype families of Cryptosporidium in bamboo rats (Rhizomys sinensis) in China. Vet Parasitol. 2015;207:144-8.
87. Wagnerová P, Sak B, McEvoy J, Rost M, Matysiak AP, Ježková J, et al. Genetic diversity of Cryptosporidium spp. including novel identification of the Cryptosporidium muris and Cryptosporidium tyzzeri in horses in the Czech Republic and Poland. Parasitol Res. 2015;114:1619-24.
88. Ondrácková Z, Kvác M, Sak B, Kvetonová D, Rost M. Prevalence and molecular characterization of Cryptosporidium spp. in dairy cattle in South Bohemia, the Czech Republic. Vet Parasitol. 2009;165:141-4.
89. Amer S, Honma H, Ikarashi M, Tada C, Fukuda Y, Suyama Y, et al. Cryptosporidium genotypes and subtypes in dairy calves in Egypt. Vet Parasitol. 2010;169:382-6.
90. Ibrahim MA, Abdel-Ghany AE, Abdel-Latef GK, Abdel-Aziz SA, Aboelhadid SM. Epidemiology and public health significance of Cryptosporidium isolated from cattle, buffaloes, and humans in Egypt. Parasitol Res. 2016;115:2439-48.

91. Helmy YA, Krücken J, Nöckler K, von Samson-Himmelstjerna G, Zessin K-H. Molecular epidemiology of Cryptosporidium in livestock animals and humans in the Ismailia province of Egypt. Vet Parasitol. 2013;193:15-24.
92. Dyachenko V, Kuhnert Y, Schmaeschke R, Etzold M, Pantchev N, Daugschies A. Occurrence and molecular characterization of *Cryptosporidium* spp. genotypes in European hedgehogs (Erinaceus europaeus L.) in Germany. Parasitology. 2010;137(02):205-16. doi: doi:10.1017/S0031182009991089.
93. Mirhashemi ME, Zintl A, Grant T, Lucy F, Mulcahy G, De Waal T. Molecular epidemiology of Cryptosporidium species in livestock in Ireland. Vet Parasitol. 2016;216:18-22.
94. Zintl A, Proctor AF, Read C, Dewaal T, Shanaghy N, Fanning S, et al. The prevalence of Cryptosporidium species and subtypes in human faecal samples in Ireland. Epidemiol Infect. 2009;137:270-7.
95. Galuppi R, Piva S, Castagnetti C, Sarli G, Iacono E, Fioravanti ML, et al. Cryptosporidium parvum: From foal to veterinary students. Vet Parasitol. 2016;219:53-6.
96. Giangaspero A, Papini R, Marangi M, Koehler AV, Gasser RB. Cryptosporidium parvum genotype IIa and Giardia duodenalis assemblage A in Mytilus galloprovincialis on sale at local food markets. Int J Food Microbiol. 2014;171:62-7.
97. Yoshida H, Matsuo M, Miyoshi T, Uchino K, Nakaguchi H, Fukumoto T, et al. An outbreak of cryptosporidiosis suspected to be related to contaminated food, October 2006, Sakai City, Japan. Jpn J Infect Dis. 2007;60:405-7.
98. Sulaiman IM, Hira PR, Zhou L, Al-Ali FM, Al-Shelahi FA, Shweiki HM, et al. Unique Endemicity of Cryptosporidiosis in Children in Kuwait. Journal of Clinical Microbiology. 2005;43(6):2805-9. doi: 10.1128/jcm.43.6.2805-2809.2005.
99. Lange H, Johansen OH, Vold L, Robertson LJ, Anthonisen IL, Nygard K. Second outbreak of infection with a rare Cryptosporidium parvum genotype in schoolchildren associated with contact with lambs/goat kids at a holiday farm in Norway. Epidemiol Infect. 2014;142:2105-13.
100. Johansen ØH, Hanevik K, Thrana F, Carlson A, Stachurska-Hagen T, Skaare D, et al. Symptomatic and asymptomatic secondary transmission of Cryptosporidium parvum following two related outbreaks in schoolchildren. Epidemiol Infect. 2015;143:1702-9.
101. Vieira PM, Mederle N, Lobo ML, Imre K, Mederle O, Xiao L, et al. Molecular characterisation of Cryptosporidium (Apicomplexa) in children and cattle in Romania. Folia Parasitol (Praha). 2014;62.
102. Imre K, Lobo LM, Matos O, Popescu C, Genchi C, Dărăbuş G. Molecular characterisation of Cryptosporidium isolates from pre-weaned calves in Romania: is there an actual risk of zoonotic infections? Vet Parasitol. 2011;181:321-4.
103. Imre K, Luca C, Costache M, Sala C, Morar A, Morariu S, et al. Zoonotic Cryptosporidium parvum in Romanian newborn lambs (Ovis aries). Vet Parasitol. 2013;191:119-22.
104. Misic Z, Abe N. Subtype analysis of Cryptosporidium parvum isolates from calves on farms around Belgrade, Serbia and Montenegro, using the 60 kDa glycoprotein gene sequences. Parasitology. 2007;134:351-8.
105. Quílez J, Torres E, Chalmers RM, Hadfield SJ, Del Cacho E, Sánchez-Acedo C. Cryptosporidium genotypes and subtypes in lambs and goat kids in Spain. Appl Environ Microbiol. 2008;74:6026-31.
106. Díaz P, Quílez J, Chalmers RM, Panadero R, López C, Sánchez-Acedo C, et al. Genotype and subtype analysis of Cryptosporidium isolates from calves and lambs in Galicia (NW Spain). Parasitology. 2010;137:1187-93.
107. Quílez J, Vergara-Castiblanco C, Monteagudo L, Del Cacho E, Sánchez-Acedo C. Multilocus fragment typing and genetic structure of Cryptosporidium parvum Isolates from diarrheic preweaned calves in Spain. Appl Environ Microbiol. 2011;77:7779-86.
108. Taylan-Ozkan A, Yasa-Duru S, Usluca S, Lysen C, Ye J, Roellig DM, et al. Cryptosporidium species and Cryptosporidium parvum subtypes in dairy calves and goat kids reared under traditional farming systems in Turkey. Exp Parasitol. 2016;170:16-20.

109. Thompson HP, Dooley JSG, Kenny J, McCoy M, Lowery CJ, Moore JE, et al. Genotypes and subtypes of Cryptosporidium spp. in neonatal calves in Northern Ireland. Parasitol Res. 2007;100:619-24.
110. Deshpande AP, Jones BL, Connelly L, Pollock KG, Brownlie S, Alexander CL. Molecular characterization of Cryptosporidium parvum isolates from human cryptosporidiosis cases in Scotland. Parasitology. 2015;142:318-25.
111. Chalmers RM, Smith RP, Hadfield SJ, Elwin K, Giles M. Zoonotic linkage and variation in Cryptosporidium parvum from patients in the United Kingdom. Parasitol Res. 2011;108:1321-5.
112. Wagnerová P, Sak B, McEvoy J, Rost M, Sherwood D, Holcomb K, et al. Cryptosporidium parvum and Enterocytozoon bieneusi in American Mustangs and Chincoteague ponies. Exp Parasitol. 2016;162:24-7.
113. Lassen B, Ståhl M, Enemark HL. Cryptosporidiosis – an occupational risk and a disregarded disease in Estonia. Acta Veterinaria Scandinavica. 2014;56(1):36. doi: 10.1186/1751-0147-56-36.
114. Kaupke A, Rzeżutka A. Emergence of novel subtypes of Cryptosporidium parvum in calves in Poland. Parasitol Res. 2015;114(12):4709-16. doi: 10.1007/s00436-015-4719-1.
115. Zhang W, Shen Y, Wang R, Liu A, Ling H, Li Y, et al. *Cryptosporidium cuniculus* and *Giardia duodenalis* in Rabbits: Genetic Diversity and Possible Zoonotic Transmission. PLoS ONE. 2012;7(2):e31262. doi: 10.1371/journal.pone.0031262.
116. Koehler AV, Whipp MJ, Haydon SR, Gasser RB. *Cryptosporidium cuniculus* - new records in human and kangaroo in Australia. Parasites & Vectors. 2014;7:492. doi: 10.1186/s13071-014-0492-8. PubMed PMID: PMC4221722.
117. Nolan MJ, Jex AR, Haydon SR, Stevens MA, Gasser RB. Molecular detection of Cryptosporidium cuniculus in rabbits in Australia. Infect Genet Evol. 2010;10:1179-87.
118. Shi K, Jian F, Lv C, Ning C, Zhang L, Ren X, et al. Prevalence, genetic characteristics, and zoonotic potential of Cryptosporidium species causing infections in farm rabbits in China. J Clin Microbiol. 2010;48:3263-6.
119. Chalmers R, Robinson G, Elwin K, Hadfield S, Xiao L, Ryan U, et al. *Cryptosporidium* sp. rabbit genotype, a newly identified human pathogen. Emerging Infectious Diseases. 2009;15(5):829-30. doi: 10.3201/eid1505.081419.
120. Robinson G, Wright S, Elwin K, Hadfield SJ, Katzer F, Bartley PM, et al. Re-description of Cryptosporidium cuniculus Inman and Takeuchi, 1979 (Apicomplexa: Cryptosporidiidae): morphology, biology and phylogeny. Int J Parasitol. 2010;40:1539-48.
121. Kaupke A, Kwit E, Chalmers RM, Michalski MM, Rzeżutka A. An outbreak of massive mortality among farm rabbits associated with Cryptosporidium infection. Res Vet Sci. 2014;97:85-7.
122. Liu X, Zhou X, Zhong Z, Chen W, Deng J, Niu L, et al. New Subtype of Cryptosporidium cuniculus Isolated from Rabbits by Sequencing the Gp60 Gene. Journal of Parasitology. 2014;100(4):532-6. doi: 10.1645/13-223.1.
123. Laatamna AE, Wagnerová P, Sak B, Květoňová D, Aissi M, Rost M, et al. Equine cryptosporidial infection associated with Cryptosporidium hedgehog genotype in Algeria. Vet Parasitol. 2013;197:350-3.
124. Kváč M, Saková K, Květoňová D, Kicia M, Wesołowska M, McEvoy J, et al. Gastroenteritis caused by the *Cryptosporidium* hedgehog genotype in an immunocompetent man. Journal of Clinical Microbiology. 2014;52(1):347-9. doi: 10.1128/jcm.02456-13.
125. Lasek-Nesselquist E, Welch DM, Thompson RC, Steuart RF, Sogin ML. Genetic exchange within and between assemblages of Giardia duodenalis. J Eukaryot Microbiol. 2009;56. doi: 10.1111/j.1550-7408.2009.00443.x.
126. Sommer MF, Beck R, Ionita M, Stefanovska J, Vasić A, Zdravković N, et al. Multilocus sequence typing of canine Giardia duodenalis from South Eastern European countries. Parasitol Res. 2015;114(6):2165-74. doi: 10.1007/s00436-015-4405-3.

127. Yang R, Jacobson C, Gardner G, Carmichael I, Campbell AJD, Ryan U. Development of a quantitative PCR (qPCR) for Giardia and analysis of the prevalence, cyst shedding and genotypes of Giardia present in sheep across four states in Australia. Exp Parasitol. 2014;137:46-52.
128. Read CM, Monis PT, Andrew Thompson RC. Discrimination of all genotypes of *Giardia duodenalis* at the glutamate dehydrogenase locus using PCR-RFLP. Infection, Genetics and Evolution. 2004;4(2):125-30. doi: http://dx.doi.org/10.1016/j.meegid.2004.02.001.
129. Asher AJ, Hose G, Power ML. Giardiasis in NSW: Identification of Giardia duodenalis assemblages contributing to human and cattle cases, and an epidemiological assessment of sporadic human giardiasis. Infect Genet Evol. 2016;44:157-61.
130. Yang R, Lee J, Ng J, Ryan U. High prevalence Giardia duodenalis assemblage B and potentially zoonotic subtypes in sporadic human cases in Western Australia. Int J Parasitol. 2010;40:293-7.
131. Zahedi A, Field D, Ryan U. Molecular typing of Giardia duodenalis in humans in Queensland - first report of Assemblage E. Parasitology. 2017:1-8.
132. Lee MF, Auer H, Lindo JF, Walochnik J. Multilocus sequence analysis of Giardia spp. isolated from patients with diarrhea in Austria. Parasitol Res. 2017;116:477-81.
133. Ulloa-Stanojlović FM, Aguiar B, Jara LM, Sato MIZ, Guerrero JA, Hachich E, et al. Occurrence of Giardia intestinalis and Cryptosporidium sp. in wastewater samples from São Paulo State, Brazil, and Lima, Peru. Environ Sci Pollut Res Int. 2016;23:22197-205.
134. Fernandes LN, de Souza PP, de Araújo RS, Razzolini MTP, Soares RM, Sato MIZ, et al. Detection of assemblages A and B of Giardia duodenalis in water and sewage from São Paulo state, Brazil. J Water Health. 2011;9:361-7.
135. Oliveira-Arbex AP, David EB, Oliveira-Sequeira TCG, Bittencourt GN, Guimarães S. Genotyping of Giardia duodenalis isolates in asymptomatic children attending daycare centre: evidence of high risk for anthroponotic transmission. Epidemiol Infect. 2016;144:1418-28.
136. Soares RM, de Souza SLP, Silveira LH, Funada MR, Richtzenhain LJ, Gennari SM. Genotyping of potentially zoonotic Giardia duodenalis from exotic and wild animals kept in captivity in Brazil. Vet Parasitol. 2011;180:344-8.
137. Colli CM, Bezagio RC, Nishi L, Bignotto TS, Ferreira ÉC, Falavigna-Guilherme AL, et al. Identical Assemblage of Giardia duodenalis in Humans, Animals and Vegetables in an Urban Area in Southern Brazil Indicates a Relationship among Them. PLoS ONE. 2015;10(3):e0118065. doi: 10.1371/journal.pone.0118065. PubMed PMID: PMC4356552.
138. Souza SLP, Gennari SM, Richtzenhain LJ, Pena HFJ, Funada MR, Cortez A, et al. Molecular identification of *Giardia duodenalis* isolates from humans, dogs, cats and cattle from the state of São Paulo, Brazil, by sequence analysis of fragments of glutamate dehydrogenase (gdh) coding gene. Veterinary Parasitology. 2007;149(3–4):258-64. doi: http://dx.doi.org/10.1016/j.vetpar.2007.08.019.
139. Fava NMN, Soares RM, Scalia LAM, Cunha MJRd, Faria ESM, Cury MC. Molecular typing of canine Giardia duodenalis isolates from Minas Gerais, Brazil. Exp Parasitol. 2016;161:1-5.
140. Scalia LAM, Fava NMN, Soares RM, Limongi JE, da Cunha MJR, Pena IF, et al. Multilocus genotyping of Giardia duodenalis in Brazilian children. Trans R Soc Trop Med Hyg. 2016;110:343-9.
141. Fava NMN, Soares RM, Scalia LAM, Kalapothakis E, Pena IF, Vieira CU, et al. Performance of Glutamate Dehydrogenase and Triose Phosphate Isomerase Genes in the Analysis of Genotypic Variability of Isolates of Giardia duodenalis from Livestocks. BioMed Research International. 2013;2013:9. doi: 10.1155/2013/875048.
142. Inpankaew T, Schär F, Odermatt P, Dalsgaard A, Chimnoi W, Khieu V, et al. Low risk for transmission of zoonotic Giardia duodenalis from dogs to humans in rural Cambodia. Parasit Vectors. 2014;7:412.
143. McDowall RM, Peregrine AS, Leonard EK, Lacombe C, Lake M, Rebelo AR, et al. Evaluation of the zoonotic potential of Giardia duodenalis in fecal samples from dogs and cats in Ontario. Can Vet J. 2011;52:1329-33.

144. Zheng G, Alsarakibi M, Liu Y, Hu W, Luo Q, Tan L, et al. Genotyping of Giardia duodenalis isolates from dogs in Guangdong, China based on multi-locus sequence. Korean J Parasitol. 2014;52:299-304.
145. Wang X, Cai M, Jiang W, Wang Y, Jin Y, Li N, et al. High genetic diversity of Giardia duodenalis assemblage E in pre-weaned dairy calves in Shanghai, China, revealed by multilocus genotyping. Parasitol Res. 2017.
146. Qi M, Cai J, Wang R, Li J, Jian F, Huang J, et al. Molecular characterization of Cryptosporidium spp. and Giardia duodenalis from yaks in the central western region of China. BMC Microbiol. 2015;15:108.
147. Qi M, Dong H, Wang R, Li J, Zhao J, Zhang L, et al. Infection rate and genetic diversity of Giardia duodenalis in pet and stray dogs in Henan Province, China. Parasitol Int. 2016;65:159-62.
148. Qi M, Wang H, Jing B, Wang R, Jian F, Ning C, et al. Prevalence and multilocus genotyping of Giardia duodenalis in dairy calves in Xinjiang, Northwestern China. Parasit Vectors. 2016;9:546.
149. Qi M, Xi J, Li J, Wang H, Ning C, Zhang L. Prevalence of Zoonotic Giardia duodenalis Assemblage B and First Identification of Assemblage E in Rabbit Fecal Samples Isolates from Central China. J Eukaryot Microbiol. 2015;62:810-4.
150. Qi M, Yu F, Li S, Wang H, Luo N, Huang J, et al. Multilocus genotyping of potentially zoonotic Giardia duodenalis in pet chinchillas (Chinchilla lanigera) in China. Vet Parasitol. 2015;208:113-7.
151. Karim MR, Wang R, Yu F, Li T, Dong H, Li D, et al. Multi-locus analysis of Giardia duodenalis from nonhuman primates kept in zoos in China: geographical segregation and host-adaptation of assemblage B isolates. Infect Genet Evol. 2015;30:82-8.
152. Wang H, Zhao G, Chen G, Jian F, Zhang S, Feng C, et al. Multilocus genotyping of Giardia duodenalis in dairy cattle in Henan, China. PLoS One. 2014;9:e100453.
153. Wang XT, Wang RJ, Ren GJ, Yu ZQ, Zhang LX, Zhang SY, et al. Multilocus genotyping of Giardia duodenalis and Enterocytozoon bieneusi in dairy and native beef (Qinchuan) calves in Shaanxi province, northwestern China. Parasitol Res. 2016;115:1355-61.
154. Zheng G, Hu W, Liu Y, Luo Q, Tan L, Li G. Occurrence and molecular identification of Giardia duodenalis from stray cats in Guangzhou, southern China. Korean J Parasitol. 2015;53:119-24.
155. Zhang X-X, Tan Q-D, Zhao G-H, Ma J-G, Zheng W-B, Ni X-T, et al. Prevalence, Risk Factors and Multilocus Genotyping of Giardia intestinalis in Dairy Cattle, Northwest China. J Eukaryot Microbiol. 2016;63:498-504.
156. Zhang X-X, Zheng W-B, Ma J-G, Yao Q-X, Zou Y, Bubu C-J, et al. Occurrence and multilocus genotyping of Giardia intestinalis assemblage C and D in farmed raccoon dogs, Nyctereutes procyonoides, in China. Parasit Vectors. 2016;9:471.
157. Wang H, Qi M, Zhang K, Li J, Huang J, Ning C, et al. Prevalence and genotyping of Giardia duodenalis isolated from sheep in Henan Province, central China. Infect Genet Evol. 2016;39:330-5.
158. Huang J, Yue D, Qi M, Wang R, Zhao J, Li J, et al. Prevalence and molecular characterization of Cryptosporidium spp. and Giardia duodenalis in dairy cattle in Ningxia, northwestern China. BMC Vet Res. 2014;10:292.
159. Zhang X-X, Zhang F-K, Li F-C, Hou J-L, Zheng W-B, Du S-Z, et al. The presence of Giardia intestinalis in donkeys, Equus asinus, in China. Parasit Vectors. 2017;10:3.
160. Santín M, Cortés Vecino JA, Fayer R. A large scale molecular study of Giardia duodenalis in horses from Colombia. Vet Parasitol. 2013;196:31-6.
161. Ramírez JD, Heredia RD, Hernández C, León CM, Moncada LI, Reyes P, et al. Molecular diagnosis and genotype analysis of *Giardia duodenalis* in asymptomatic children from a rural area in central Colombia. Infection, Genetics and Evolution. 2015;32:208-13. doi: http://dx.doi.org/10.1016/j.meegid.2015.03.015.
162. Beck R, Sprong H, Pozio E, Cacciò SM. Genotyping Giardia duodenalis Isolates from Dogs: Lessons from a Multilocus Sequence Typing Study. Vector-Borne and Zoonotic Diseases. 2011;12(3):206-13. doi: 10.1089/vbz.2011.0751.

163. Pelayo L, Nuñez FA, Rojas L, Furuseth Hansen E, Gjerde B, Wilke H, et al. Giardia infections in Cuban children: the genotypes circulating in a rural population. Ann Trop Med Parasitol. 2008;102:585-95.
164. Atherton R, Bhavnani D, Calvopiña M, Vicuña Y, Cevallos W, Eisenberg J. Molecular identification of Giardia duodenalis in Ecuador by polymerase chain reaction-restriction fragment length polymorphism. Mem Inst Oswaldo Cruz. 2013;108:512-5.
165. Fahmy HM, El-Serougi AO, El Deeb HK, Hussein HM, Abou-Seri HM, Klotz C, et al. Giardia duodenalis assemblages in Egyptian children with diarrhea. Eur J Clin Microbiol Infect Dis. 2015;34:1573-81.
166. Soliman RH, Fuentes I, Rubio JM. Identification of a novel Assemblage B subgenotype and a zoonotic Assemblage C in human isolates of Giardia intestinalis in Egypt. Parasitol Int. 2011;60:507-11.
167. Wegayehu T, Karim MR, Erko B, Zhang L, Tilahun G. Multilocus genotyping of Giardia duodenalis isolates from calves in Oromia Special Zone, Central Ethiopia. Infect Genet Evol. 2016;43:281-8.
168. Wegayehu T, Karim MR, Li J, Adamu H, Erko B, Zhang L, et al. Multilocus genotyping of Giardia duodenalis isolates from children in Oromia Special Zone, central Ethiopia. BMC Microbiol. 2016;16:89.
169. Sotiriadou I, Pantchev N, Gassmann D, Karanis P. Molecular identification of Giardia and Cryptosporidium from dogs and cats. Parasite. 2013;20:8.
170. Anim-Baidoo I, Narh CA, Oddei D, Brown CA, Enweronu-Laryea C, Bandoh B, et al. Giardia lamblia infections in children in Ghana. Pan Afr Med J. 2015;24:217.
171. Laishram S, Kannan A, Rajendran P, Kang G, Ajjampur SSR. Mixed Giardia duodenalis assemblage infections in children and adults in South India. Epidemiol Infect. 2012;140:2023-7.
172. Broglia A, Weitzel T, Harms G, Cacció SM, Nöckler K. Molecular typing of *Giardia duodenalis* isolates from German travellers. Parasitol Res. 2013;112(10):3449-56. doi: 10.1007/s00436-013-3524-y.
173. Sarkari B, Ashrafmansori A, Hatam GR, Motazedian MH, Asgari Q, Mohammadpour I. Genotyping of Giardia lamblia isolates from human in southern Iran. Trop Biomed. 2012;29:366-71.
174. Rafiei A, Roointan ES, Samarbafzadeh AR, Shayesteh AA, Shamsizadeh A, Pourmahdi Borujeni M. Investigation of Possible Correlation between Giardia duodenalis Genotypes and Clinical Symptoms in Southwest of Iran. Iran J Parasitol. 2013;8:389-95.
175. Hatam-Nahavandi K, Mohebali M, Mahvi A-H, Keshavarz H, Mirjalali H, Rezaei S, et al. Subtype analysis of Giardia duodenalis isolates from municipal and domestic raw wastewaters in Iran. Environ Sci Pollut Res Int. 2017;24:12740-7.
176. De Liberato C, Berrilli F, Marangi M, Santoro M, Trogu T, Putignani L, et al. Giardia duodenalis in Alpine (Rupicapra rupicapra rupicapra) and Apennine (Rupicapra pyrenaica ornata) chamois. Parasites & Vectors. 2015;8(1):650. doi: 10.1186/s13071-015-1243-1.
177. Lee MF, Cadogan P, Eytle S, Copeland S, Walochnik J, Lindo JF. Molecular epidemiology and multilocus sequence analysis of potentially zoonotic Giardia spp. from humans and dogs in Jamaica. Parasitol Res. 2016;116:409-14.
178. Itagaki T, Kinoshita S, Aoki M, Itoh N, Saeki H, Sato N, et al. Genotyping of Giardia intestinalis from domestic and wild animals in Japan using glutamete dehydrogenase gene sequencing. Vet Parasitol. 2005;133:283-7.
179. Abe N, Kimata I, Iseki M. Identification of genotypes of Giardia intestinalis isolates from dogs in Japan by direct sequencing of the PCR amplified glutamate dehydrogenase gene. J Vet Med Sci. 2003;65:29-33.
180. Abe N, Tanoue T, Noguchi E, Ohta G, Sakai H. Molecular characterization of Giardia duodenalis isolates from domestic ferrets. Parasitol Res. 2010;106:733-6.

181. Abe N, Teramoto I. Molecular evidence for person-to-person transmission of a novel subtype in Giardia duodenalis assemblage B at the rehabilitation institution for developmentally disabled people. Parasitol Res. 2012;110:1025-8.
182. Itoh N, Itagaki T, Kawabata T, Konaka T, Muraoka N, Saeki H, et al. Prevalence of intestinal parasites and genotyping of Giardia intestinalis in pet shop puppies in east Japan. Vet Parasitol. 2011;176:74-8.
183. Suzuki J, Murata R, Kobayashi S, Sadamasu K, Kai A, Takeuchi T. Risk of human infection with *Giardia duodenalis* from cats in Japan and genotyping of the isolates to assess the route of infection in cats. Parasitology. 2011;138(04):493-500. doi: doi:10.1017/S0031182010001459.
184. Hijjawi N, Yang R, Mukbel R, Yassin Y, Mharib T, Ryan U. First genetic characterisation of Giardia in human isolates from Jordan. Parasitol Res. 2016;115:3723-9.
185. Mbae C, Mulinge E, Guleid F, Wainaina J, Waruru A, Njiru ZK, et al. Molecular Characterization of Giardia duodenalis in Children in Kenya. BMC Infect Dis. 2016;16:135.
186. Huey CS, Mahdy MAK, Al-Mekhlafi HM, Nasr NA, Lim YAL, Mahmud R, et al. Multilocus genotyping of Giardia duodenalis in Malaysia. Infect Genet Evol. 2013;17:269-76.
187. Torres-Romero JC, Euan-Canto AdJ, Benito-González N, Padilla-Montaño N, Huchin-Chan C, Lara-Riegos J, et al. Intestinal parasites and genotyping of Giardia duodenalis in children: first report of genotype B in isolates from human clinical samples in Mexico. Mem Inst Oswaldo Cruz. 2014;109:388-90.
188. El Fatni C, Olmo F, El Fatni H, Romero D, Rosales MJ. First genotyping of Giardia duodenalis and prevalence of enteroparasites in children from Tetouan (Morocco). Parasite. 2013;21:48.
189. van der Giessen JWB, de Vries A, Roos M, Wielinga P, Kortbeek LM, Mank TG. Genotyping of Giardia in Dutch patients and animals: a phylogenetic analysis of human and animal isolates. Int J Parasitol. 2006;36:849-58.
190. Lebbad M, Ankarklev J, Tellez A, Leiva B, Andersson JO, Svärd S. Dominance of Giardia assemblage B in León, Nicaragua. Acta Trop. 2008;106:44-53.
191. Robertson LJ, Forberg T, Hermansen L, Hamnes IS, Gjerde B. Giardia duodenalis cysts isolated from wild moose and reindeer in Norway: genetic characterization by PCR-rflp and sequence analysis at two genes. J Wildl Dis. 2007;43:576-85.
192. Hussein AIA, Yamaguchi T, Nakamoto K, Iseki M, Tokoro M. Multiple-subgenotype infections of Giardia intestinalis detected in Palestinian clinical cases using a subcloning approach. Parasitol Int. 2009;58:258-62.
193. Peréz Cordón G, Cordova Paz Soldan O, Vargas Vásquez F, Velasco Soto JR, Sempere Bordes L, Sánchez Moreno M, et al. Prevalence of enteroparasites and genotyping of Giardia lamblia in Peruvian children. Parasitol Res. 2008;103:459-65.
194. Solarczyk P, Majewska AC. Prevalence and multilocus genotyping of Giardia from animals at the zoo of Poznan, Poland. Wiad Parazytol. 2011;57:169-73.
195. Solarczyk P, Majewska AC, Moskwa B, Cabaj W, Dabert M, Nowosad P. Multilocus genotyping of Giardia duodenalis isolates from red deer (Cervus elaphus) and roe deer (Capreolus capreolus) from Poland. Folia Parasitol (Praha). 2012;59:237-40.
196. Adriana G, Zsuzsa K, Mirabela Oana D, Mircea GC, Viorica M. Giardia duodenalis genotypes in domestic and wild animals from Romania identified by PCR-RFLP targeting the gdh gene. Vet Parasitol. 2016;217:71-5.
197. Lalle M, Bruschi F, Castagna B, Campa M, Pozio E, Cacciò SM. High genetic polymorphism among Giardia duodenalis isolates from Sahrawi children. Trans R Soc Trop Med Hyg. 2009;103:834-8.
198. Soba B, Islamovic S, Skvarc M, Caccio SM. Multilocus genotyping of Giardia duodenalis (Lambl, 1859) from symptomatic human infections in Slovenia. Folia Parasitol (Praha). 2015;62.
199. Fernández-Álvarez Á, Martín-Alonso A, Abreu-Acosta N, Feliu C, Hugot J-P, Valladares B, et al. Identification of a novel assemblage G subgenotype and a zoonotic assemblage B in rodent isolates of Giardia duodenalis in the Canary Islands, Spain. Parasitology. 2014;141:206-15.

200. de Lucio A, Bailo B, Aguilera M, Cardona GA, Fernández-Crespo JC, Carmena D. No molecular epidemiological evidence supporting household transmission of zoonotic Giardia duodenalis and Cryptosporidium spp. from pet dogs and cats in the province of Álava, Northern Spain. Acta Trop. 2017;170:48-56.
201. de Lucio A, Martínez-Ruiz R, Merino FJ, Bailo B, Aguilera M, Fuentes I, et al. Molecular Genotyping of Giardia duodenalis Isolates from Symptomatic Individuals Attending Two Major Public Hospitals in Madrid, Spain. PLoS One. 2015;10:e0143981.
202. Gómez-Muñoz MT, Cámara-Badenes C, Martínez-Herrero MdC, Dea-Ayuela MA, Pérez-Gracia MT, Fernández-Barredo S, et al. Multilocus genotyping of Giardia duodenalis in lambs from Spain reveals a high heterogeneity. Res Vet Sci. 2012;93:836-42.
203. Martínez-Díaz RA, Sansano-Maestre J, Martínez-Herrero MDC, Ponce-Gordo F, Gómez-Muñoz MT. Occurrence and genetic characterization of Giardia duodenalis from captive nonhuman primates by multi-locus sequence analysis. Parasitol Res. 2011;109:539-44.
204. Cardona GA, de Lucio A, Bailo B, Cano L, de Fuentes I, Carmena D. Unexpected finding of feline-specific Giardia duodenalis assemblage F and Cryptosporidium felis in asymptomatic adult cattle in Northern Spain. Vet Parasitol. 2015;209:258-63.
205. Lebbad M, Petersson I, Karlsson L, Botero-Kleiven S, Andersson JO, Svenungsson B, et al. Multilocus genotyping of human Giardia isolates suggests limited zoonotic transmission and association between assemblage B and flatulence in children. PLoS Negl Trop Dis. 2011;5:e1262.
206. Di Cristanziano V, Santoro M, Parisi F, Albonico M, Shaali MA, Di Cave D, et al. Genetic characterization of Giardia duodenalis by sequence analysis in humans and animals in Pemba Island, Tanzania. Parasitol Int. 2014;63:438-41.
207. Siripattanapipong S, Leelayoova S, Mungthin M, Thompson RCA, Boontanom P, Saksirisampant W, et al. Clonal diversity of the glutamate dehydrogenase gene in *Giardia duodenalis* from Thai Isolates: evidence of genetic exchange or Mixed Infections? BMC Microbiology. 2011;11:206-. doi: 10.1186/1471-2180-11-206. PubMed PMID: PMC3191338.
208. Siripattanapipong S, Leelayoova S, Mungthin M, Thompson RCA, Boontanom P, Saksirisamphant W, et al. Determination of discriminatory power of genetic markers used for genotyping Giardia duodenalis. Southeast Asian J Trop Med Public Health. 2011;42:764-71.
209. Koloren Z, Seferoğlu O, Karanis P. Occurency of Giardia duodenalis assemblages in river water sources of Black Sea, Turkey. Acta Trop. 2016;164:337-44.
210. Johnston AR, Gillespie TR, Rwego IB, Tranby McLachlan TL, Kent AD, Goldberg TL. Molecular Epidemiology of Cross-Species Giardia duodenalis Transmission in Western Uganda. PLOS Neglected Tropical Diseases. 2010;4(5):e683. doi: 10.1371/journal.pntd.0000683.
211. Scorza AV, Ballweber LR, Tangtrongsup S, Panuska C, Lappin MR. Comparisons of mammalian Giardia duodenalis assemblages based on the β-giardin, glutamate dehydrogenase and triose phosphate isomerase genes. Veterinary Parasitology. 2012;189(2–4):182-8. doi: http://dx.doi.org/10.1016/j.vetpar.2012.04.032.
212. Wang A, Ruch-Gallie R, Scorza V, Lin P, Lappin MR. Prevalence of Giardia and Cryptosporidium species in dog park attending dogs compared to non-dog park attending dogs in one region of Colorado. Vet Parasitol. 2012;184:335-40.
213. Lasek-Nesselquist E, Welch DM, Sogin ML. The identification of a new *Giardia duodenalis* assemblage in marine vertebrates and a preliminary analysis of *G. duodenalis* population biology in marine systems. International Journal for Parasitology. 2010;40(9):1063-74. doi: http://dx.doi.org/10.1016/j.ijpara.2010.02.015.
214. Minetti C, Lamden K, Durband C, Cheesbrough J, Fox A, Wastling JM. Determination of Giardia duodenalis assemblages and multi-locus genotypes in patients with sporadic giardiasis from England. Parasites & Vectors. 2015;8(1):444. doi: 10.1186/s13071-015-1059-z.
215. Langkjær RB, Vigre H, Enemark HL, Maddox-Hyttel C. Molecular and phylogenetic characterization of Cryptosporidium and Giardia from pigs and cattle in Denmark. Parasitology. 2007;134(03):339-50. doi: doi:10.1017/S0031182006001533.
